# Wheat Bioactive Compounds and Human Health: A Review of Nutraceutical Potential, Molecular Mechanisms and AI-Assisted Functional Food Innovation

**DOI:** 10.3390/ijms27188290

**Published:** 2026-09-17

**Authors:** Ramachandran Vinayagam, Kumar Ganesan, Hwang Bae Sohn, Myoung-Goo Choi, Youn-Il Park, Awdhesh Kumar Mishra, Kwang-Hyun Baek

**Affiliations:** 1 Department of Biotechnology, College of Sciences, Yeungnam University, 280 Daehak-ro, Gyeongsan-si 38541, Republic of Korea; awdhesh@ynu.ac.kr; 2 School of Chinese Medicine, Li Ka Shing Faculty of Medicine, The University of Hong Kong, Pokfulam, Hong Kong SAR, China; kumarg@hku.hk; 3 National Institute of Crop Science, Rural Development Administration, Wanju-Gun 55365, Republic of Korea; mission7@korea.kr (H.B.S.); cmg0305@korea.kr (M.-G.C.); 4 Department of Biological Sciences, Chungnam National University, Daejeon 34134, Republic of Korea; yipark@cnu.ac.kr

**Keywords:** wheat, functional foods, bioactive compounds, artificial intelligence, nutraceuticals, metabolic health

## Abstract

Wheat (*Triticum aestivum* L.) is among the most widely consumed cereal grains worldwide and is essential for global food security. This review critically examines current evidence on wheat bioactive compounds, nutritional composition, and health-promoting properties. Wheat contains diverse nutritional and bioactive compounds, including phenolic acids, flavonoids, lignans, alkylresorcinols, phytosterols, and bioactive peptides. This review systematically evaluates evidence across multiple levels, from chemical characterization and cell-based studies to animal models and human clinical trials, with explicit differentiation of evidence quality. Wheat-derived compounds demonstrate antioxidant, antidiabetic, anti-obesity, anti-inflammatory, anticancer, antimicrobial, and gut microbiota-modulating activities in preclinical models. Identified molecular mechanisms converge on Nrf2-mediated antioxidant defense, NF-κB inflammatory pathway inhibition, AMPK metabolic regulation, and PI3K/AKT insulin signaling. Recent advances in artificial intelligence (AI) and machine learning provide new tools for identifying wheat bioactive molecules, predicting biological targets, and assessing flour quality through hyperspectral imaging. AI-integrated multi-omics approaches offer potential for personalized wheat-based nutrition strategies by matching individual genetic, metabolic, and microbiome profiles to specific wheat varieties or bioactive extracts. This review concludes that wheat-derived bioactive compounds show significant nutraceutical potential; however, clinical translation requires rigorous human studies, and safety considerations for gluten-sensitive populations must be addressed.

## 1. Introduction

Cereal foods are rich sources of proteins, dietary fiber, carbohydrates, macronutrients, micronutrients (vitamins and minerals), and bioactive compounds with pharmacological activity [1]. Higher whole-grain cereal intake is associated with a reduced disease risk and health benefits beyond basic nutrition [2]. Cereal-based functional foods protect against oxidative stress and chronic inflammation. They reduce the risk of chronic degenerative diseases and certain cancers [3].

Metabolic syndrome (MetS) comprises physical and metabolic disorders, including insulin resistance, obesity, hypertension, dyslipidemia, and diabetes mellitus (DM). These conditions are also associated with insufficient physical activity and sedentary lifestyles [4]. Functional cereal components are crucial for glucose homeostasis by reducing postprandial glucose spikes, improving insulin sensitivity, and reducing the risk of DM [5]. Bioactive compounds in whole grains, including wheat, barley, and oats, regulate blood glucose levels through multiple mechanisms. These whole grains are high-fiber, low-glycemic-index foods [6,7]. Numerous studies consistently show that replacing some animal-derived lipids and proteins with cereal foods improves health in patients with MetS. Specifically, whole grains reduce disease risk and mortality [8,9].

Wheat (*Triticum aestivum* L.) is among the most valuable staple crops worldwide, belonging to the Poaceae family and providing essential nutrition for over one-third of the global population. The most widely cultivated species is *T. aestivum* (bread wheat), followed by *T. durum* (durum wheat), which is commonly used for bread, pasta, and other foods. The wheat grain consists of three major anatomical parts: bran (rich in fiber and phenolic compounds), germ (containing lipids and vitamins), and endosperm (containing starch and proteins) [10]. As a major source of carbohydrates, protein, fiber, and micronutrients, wheat provides approximately 20% of global calorie and protein intake [10]. Beyond its nutritional value, whole wheat contains the highest levels of phenolic compounds, providing greater nutraceutical benefits than refined wheat products. Wheat contains high levels of phenolic compounds and dietary fiber, which act as antioxidants, inhibit low-density lipoprotein oxidation and DNA damage [11], and reduce the risk of type 2 diabetes (T2D) and obesity while improving inflammatory profiles [12,13]. Therefore, wheat and its products are increasingly explored as functional foods with potential biological applications.

The primary objective of this review is to systematically evaluate the nutraceutical potential of wheat bioactive compounds with explicit evidence-level classification across compositional, cell-based, animal, and human studies. The secondary objective is to synthesize the integrated molecular mechanisms underlying the health effects of wheat-derived compounds across multiple disease contexts. The tertiary objective is to critically assess emerging artificial intelligence (AI)-assisted approaches for wheat chemical profiling, bioactive compound discovery, and functional food innovation. The final objective is to identify unresolved scientific questions and propose evidence-based future research directions for translating wheat bioactive compounds into clinical and functional food applications.

### 1.1. Novelty and Scope of This Review

While previous reviews have comprehensively addressed wheat phytochemical composition [12,13,14] or specific health outcomes [15,16], this review provides a uniquely integrated perspective across four novel dimensions. First, present a systematic comparative analysis of bioactive compound distribution across all grain fractions (bran, aleurone, germ, endosperm, testa, and pericarp) with quantitative data enabling direct comparisons. Second, comprehensively cover the full spectrum of health benefits with mechanistic details across cellular, animal, and human studies, including critical evaluation of gluten-related safety considerations. Third, synthesize emerging evidence on bioprocessing strategies (germination, fermentation, and enzymatic hydrolysis) that enhance bioactive compound release and bioactivity. Fourth, provide a dedicated review of artificial intelligence (AI)-assisted approaches for wheat chemical profiling, nutraceutical discovery, quality prediction, and personalized nutrition. This integrated framework positions wheat not merely as a staple crop but as a versatile platform for next-generation functional food innovation and precision healthcare applications.

### 1.2. Review Methodology

The literature search was performed using PubMed, Scopus, Web of Science, and Google Scholar databases. Search strings included combinations of the following terms: “wheat,” “*Triticum aestivum*,” “bioactive compounds,” “phenolic acids,” “flavonoids,” “antioxidant,” “antidiabetic,” “anti-obesity,” “anti-inflammatory,” “anticancer,” “gut microbiota,” “artificial intelligence,” “machine learning,” and “functional foods.” Eligibility criteria included original research articles, reviews, and clinical studies. Studies were included if they addressed wheat bioactive compounds, health effects, or AI-assisted analysis. Exclusion criteria included non-English publications, conference abstracts, and studies on wheat-related topics without bioactive compound or health outcome data. The screening process involved title and abstract review followed by full-text assessment. Data extraction captured study design, wheat material, bioactive compounds, experimental model, dose/duration, outcomes, and limitations. Evidence was classified into six levels: Level 1 (randomized controlled trials), Level 2 (observational human studies), Level 3 (animal studies), Level 4 (cell-based experiments), Level 5 (chemical/analytical studies), and Level 6 (computational predictions).

## 2. Bioactive Compounds in Wheat

### 2.1. Phenolic Compounds

Phenolic acids are secondary metabolites that contribute to the bioactive profile of plants and are central to the antioxidant and health benefits of wheat [12]. They are generally classified into two subgroups: hydroxybenzoic acid (HBA) and hydroxycinnamic acid (HCA) derivatives. HBA derivatives include syringic acid, p-hydroxybenzoic acid, vanillic acid, and protocatechuic acid. HCA derivatives include ferulic acid, caffeic acid, p-coumaric acid, and sinapic acid [12,13]. Okarter et al. reported a total phenolic content of 53–69% across six wheat varieties [14].

Ancient wheat genotypes contain higher levels of syringic acid and tyrosol. These genotypes also exhibit higher total phenolic content and higher concentrations of resorcinol, tyrosol, caffeic acid, syringic acid, and ferulic acid compared to modern varieties [15]. Whole-grain wheat flour has the highest total phenolic content at 1.53 mg gallic acid equivalent per gram (GAE/g), followed by white wheat flour at 1.20 mg GAE/g [16]. Genotype and growing location significantly affect phenolic content and antioxidant capacity in *T. durum* [17].

Colored wheat varieties (blue, purple, and black) are rich in phenolic compounds and flavonoid pigments. Soluble and insoluble phenolic contents generally increase in the order of non-colored, purple, blue, and black wheat. Colored wheat grains may contain several-fold higher phenolic concentrations than non-colored grains [1,18,19,20]. Purple and red cereal pigmentation is attributed to proanthocyanidins, flavonoids, phenolic acids, and lignins [21].

Ferulic acid is mainly present in bound form, ester-linked to cell wall arabinoxylans in the aleurone layer and pericarp. Purple bran varieties generally exhibit higher bioactive phenolic concentrations, particularly ferulic acid and other HCAs, resulting in greater antioxidant capacity [22]. The total phenolic content of purple wheat shows substantial genotype-dependent variation. Gallic acid was the most abundant compound, ranging from 15.41 to 95.28 μg/g dry weight. Protocatechuic acid and ellagic acid ranged from 6.93 to 66.35 and 8.20 to 19.64 μg/g dry weight, respectively. Ferulic acid and sinapic acid reached exceptionally high levels of 581.96 and 277–619 μg/g dry weight, respectively, in certain purple wheat genotypes [23].

Phenolic acids in wheat grains mainly occur as insoluble bound forms (50–70%) linked to cell wall components such as arabinoxylan, cellulose, lignin, and proteins. Smaller amounts occur as soluble conjugated (13–20%) and free forms (0.5–2%) [24]. Ferulic acid is the predominant phenolic compound, accounting for approximately 70–90% of total phenolic acids in wheat grain. The seven phenolic acids identified in wheat include p-hydroxybenzoic acid, hydroxytyrosol, vanillic acid, syringic acid, trans-ferulic acid, sinapic acid, trans-caffeic acid, and trans-p-coumaric acid [25]. Ferulic acid represents 65.0–94.9% of insoluble bound phenolic compounds [26]. The soluble phenolic fraction of sprouted wheat mainly comprises HCAs with minimal HBAs and flavonoids [27].

Wheat germ is a nutritionally valuable fraction with a phenolic profile distinct from that of bran. Wheat germ represents approximately 2–3% of kernel weight and is rich in phytochemicals, bioactive peptides, polyunsaturated fatty acids, dietary fiber, B vitamins, γ-tocopherol, phytosterols, and minerals. The germ contains carbohydrates, primarily sucrose (56–78 g/100 g dry weight), and protein (11.2–30.0 g/100 g dry weight) [28,29,30,31,32]. However, wheat germ has limited oxidative stability because of its high lipid and enzyme contents. Wheat germ also contains anti-nutritional factors such as phytic acid, raffinose, and wheat germ agglutinin [33].

Recent studies indicate that sprouted wheat flour improves the phenolic profile and bread-making performance [34]. Bioprocessed wheat ingredients differ in phenolic bioaccessibility during enzymatic digestion involving beta-glucosidases, esterases, and xylanases. Free ferulic acid increases in sprouted wheat during digestion, and phenolic content varies substantially among wheat types [27]. Table S1 compares phenolic acid contents across wheat varieties. These differences have important implications for selecting wheat varieties for functional food and nutraceutical applications.

#### Quantitative Distribution of Phenolic Compounds Across Grain Fractions

A detailed quantitative comparison reveals substantial heterogeneity in phenolic compound distribution across wheat grain fractions. The bran fraction contains the highest phenolic concentration (50–70% of total grain phenolics), followed by the germ (20–30%), while the starchy endosperm, the main component of refined white flour, contains comparatively low levels (<5%) [24]. Ferulic acid is the predominant phenolic compound in all fractions but exhibits distinct distribution patterns: the outer pericarp contains 75–93% of total ferulic acid in insoluble bound form, while the aleurone layer contains 45–65% bound ferulic acid ester-linked to arabinoxylan [24,26]. The aleurone layer is particularly enriched in HCA derivatives, including p-coumaric acid (45–120 μg/g), sinapic acid (30–85 μg/g), and caffeic acid (15–40 μg/g), which are largely absent from the endosperm [35,36]. The germ, while comprising only 2–3% of kernel weight, contributes disproportionately to the soluble phenolic fraction, with free ferulic acid (60–180 μg/g), vanillic acid (20–45 μg/g), and syringic acid (15–35 μg/g) [28,29,30]. This fraction-specific distribution has important implications: the predominance of bound phenolics in bran and pericarp necessitates colonic fermentation for bioactivation, whereas germ phenolics are more readily bioavailable in the upper gastrointestinal tract.

### 2.2. Flavonoids

Wheat flavonoids include flavones (apigenin, luteolin, and tricin), flavonols (kaempferol and quercetin), and anthocyanins (a water-soluble subclass of flavonoids). These compounds contribute substantially to antioxidant and anti-inflammatory activities. Tricin is a flavonoid of particular interest in durum wheat because of its anti-inflammatory and chemopreventive activities [37]. Pigment accumulation in the pericarp (purple) and aleurone layer (blue) is genetically controlled. Black wheat combines both traits and consequently has the highest anthocyanin content [1,38]. Anthocyanins are responsible for pigmentation in purple, blue, and black wheat varieties. The main identified anthocyanins are cyanidin-3-O-glucoside, delphinidin-3-O-glucoside, and pelargonidin derivatives [20,38].

Black wheat is rich in protein, fiber, calcium, vitamin K, flavonoids, phenolics, and antioxidant activity. Approximately 225 flavonoid-related metabolites differentiate black wheat from other varieties. These differences are associated with distinct coloration and gene expression, including TaCHI, TaDFR, TaF3H, TaUGT, TaANS, and TaMT. Whole black wheat intake is associated with reduced cardiometabolic risk factors and improved colorectal health [39].

#### Comparative Flavonoid Profiles: Colored Versus Common Wheat

The flavonoid profiles of colored wheat varieties (purple, blue, black) differ substantially from common wheat in both composition and concentration. Common wheat contains primarily flavones (apigenin, luteolin, tricin) and flavonols (kaempferol, quercetin) at total flavonoid concentrations of 150–350 μg/g dry weight [21]. In contrast, colored wheat varieties exhibit several-fold higher flavonoid concentrations and contain anthocyanins largely absent from common wheat. Purple wheat is characterized by cyanidin-3-O-glucoside (350–850 μg/g), peonidin-3-O-glucoside (120–280 μg/g), and malvidin derivatives, with total anthocyanin content ranging from 450 to 1200 μg/g depending on genotype [20,37]. Blue wheat accumulates delphinidin-3-O-glucoside (400–900 μg/g) and petunidin-3-O-glucoside (180–350 μg/g), while black wheat, which combines both purple and blue pigmentation traits, exhibits the highest total anthocyanin content (650–1450 μg/g) [1,37,40]. Beyond anthocyanins, colored wheat varieties also show elevated levels of other flavonoids. Black wheat contains approximately 225 flavonoid-related metabolites that differentiate it from common wheat, including higher concentrations of flavone C-glycosides (luteolin-6-C-glucoside, apigenin-6-C-glucoside) and flavonol O-glycosides (quercetin-3-O-glucoside, kaempferol-3-O-glucoside) [39]. These compositional differences are genetically controlled, with key regulatory genes including TaCHI, TaDFR, TaF3H, TaUGT, TaANS, and TaMT showing differential expression in colored wheat varieties [1].

### 2.3. Lignans and Alkylresorcinols

Wheat contains lignans, mainly secoisolariciresinol and syringaresinol. These compounds exhibit phytoestrogenic activity and are converted by gut microbiota into mammalian lignans, including enterodiol and enterolactone [41]. Alkylresorcinols are a unique class of phenolic lipids concentrated almost exclusively in wheat and rye bran. They are widely used as biomarkers of whole-grain intake in nutritional epidemiology [12]. Benzoxazinoid and alkylresorcinol contents in spring and winter wheat grains vary significantly with the production system [42]. In wheat, germination temperature and duration positively influence the accumulation of soluble phenolic acids, flavone C-glycosides, and lignans, thereby improving bioactive potential [27]. Wheat bran polysaccharides and fermented derivatives demonstrate strong free radical scavenging capacity and protection against oxidative damage, including inhibiting lipid peroxidation and reducing intracellular reactive oxygen species (ROS) [43,44].

### 2.4. Phytosterols

Phytosterols are plant-derived sterols that structurally resemble cholesterol and are present in wheat at concentrations ranging from 500 to 1200 μg/g dry weight, depending on variety and grain fraction [45,46,47]. The major phytosterols identified in wheat include β-sitosterol (250–600 μg/g), campesterol (80–200 μg/g), and stigmasterol (30–80 μg/g) [46]. The bran and germ fractions contain the highest phytosterol concentrations, while the endosperm contains substantially lower levels. Phytosterols exert cholesterol-lowering effects through competitive inhibition of intestinal cholesterol absorption, leading to reduced serum low-density lipoprotein cholesterol concentrations [48]. These compounds also exhibit anti-inflammatory properties through modulation of immune cell function and cytokine production. The germ fraction is particularly enriched in phytosterols, contributing to the cardioprotective effects associated with whole-grain wheat consumption [46].

### 2.5. Bioaccessibility, Bioavailability, and Metabolism of Wheat Bioactive Compounds

The health effects of wheat bioactive compounds depend not only on their concentration in the grain but also on their bioaccessibility (release from the food matrix during digestion), bioavailability (absorption and systemic distribution), and metabolic fate. Most wheat phenolics (50–70%) occur as insoluble bound forms ester-linked to cell wall arabinoxylans [24]. These bound phenolics are not released during upper gastrointestinal digestion and require colonic fermentation by gut microbiota for bioactivation. Microbial esterases hydrolyze the ester bonds, releasing free phenolic acids that can exert local antioxidant effects in the colon or be absorbed systemically [27,41]. The bioaccessibility of wheat phenolics is influenced by food processing, with germination, fermentation, and enzymatic hydrolysis enhancing release of bound forms [27,49,50].

Anthocyanins, a flavonoid subclass, are relatively unstable and undergo extensive degradation during digestion, with less than 1–2% of ingested anthocyanins typically absorbed intact. However, their colonic metabolites (phenolic acids and aldehydes) may contribute to systemic effects [40]. Flavonoids (apigenin, luteolin, tricin, kaempferol, quercetin) are absorbed to varying degrees depending on their glycosylation patterns, with aglycones showing higher bioavailability than glycosides [41].

Alkylresorcinols are absorbed intact and have a half-life of approximately 5 h in humans, making them suitable biomarkers of whole-grain intake. Lignans are converted by gut microbiota to enterodiol and enterolactone, which are absorbed and exert phytoestrogenic effects [41]. Ferulic acid, the predominant wheat phenolic, is poorly absorbed in its bound form but shows higher bioavailability after release by enzymatic or microbial hydrolysis. Absorbed ferulic acid undergoes phase II metabolism (glucuronidation and sulfation) and has a plasma half-life of approximately 2–3 h [26]. Factors affecting bioavailability include food matrix effects, processing, interactions with dietary components (fiber, proteins), and individual variation in gut microbiota composition [27,51].

The predominance of bound phenolics in wheat bran highlights the importance of colonic fermentation in mediating their bioactivity. Inter-individual variation in gut microbiota composition significantly affects phenolic metabolism and the production of bioactive metabolites, which may contribute to the variable health responses to wheat-based interventions observed in clinical studies [51].

## 3. Nutritional Composition of Wheat

Wheat is a nutrient-dense grain providing essential macro- and micronutrients. The nutritional composition varies significantly among grain fractions, with the bran and germ containing substantially higher concentrations of bioactive compounds, vitamins, and minerals compared to the starchy endosperm. The bran fraction contains the highest phenolic concentration (50–70% of total), followed by the germ (20–30%), while the starchy endosperm contains comparatively low levels (<5%) [24]. Wheat phenolic compounds exist in three forms: free, soluble conjugated (esterified to sugars or small molecules), and insoluble bound (covalently bound to cell wall polysaccharides). The bound form predominates, representing more than 75% of total phenolic content [12,13]. Pigmented durum and bread wheat contain 1.6–8.3-fold higher phenolic content and 4.0–9.3-fold higher antioxidant activity than wholemeal and refined flour, respectively [45].

Two winter wheat cultivars, Aurelius and Activus, grown in southwestern Poland, exhibit distinct nutritional profiles. Activus has higher protein, fiber, ash, and gross energy contents, while Aurelius contains higher levels of essential amino acids and beneficial macro- and microelements, indicating greater biological protein value [46]. Table 1 provides a detailed breakdown of bioactive compound distribution across wheat kernel fractions.

### 3.1. Amino Acid Composition and Nutritional Quality

Wheat proteins are characterized by their unique amino acid profile, which has important nutritional implications. The protein content of wheat ranges from 8% to 15% depending on variety, growing conditions, and grain fraction [47,53]. The major protein fractions include gliadins and glutenins (collectively gluten), which constitute approximately 80% of total wheat protein, along with albumins and globulins [53,54].

The essential amino acid profile of wheat proteins reveals lysine as the first limiting amino acid, followed by threonine. Table S2 presents the comparative amino acid composition of wheat proteins relative to animal protein sources.

The lysine deficiency in wheat proteins has important metabolic implications. Lysine is an essential amino acid required for protein synthesis, calcium absorption, and carnitine production. When lysine is limiting, the absorption and utilization of other essential amino acids is compromised, as protein synthesis requires all amino acids to be present simultaneously [55]. This concept of the “limiting amino acid” explains why wheat proteins have a lower Protein Digestibility-Corrected Amino Acid Score (PDCAAS) of approximately 0.4–0.5 compared to animal proteins (egg: 1.0, milk: 1.0, beef: 0.92) [55].

The practical implication is that wheat proteins should be complemented with lysine-rich protein sources to achieve optimal amino acid balance. Legumes (particularly pulses), which are rich in lysine but limited in methionine, complement wheat proteins effectively. This complementary relationship explains the nutritional basis for traditional food combinations such as wheat bread with beans or dal with chapatti. The PDCAAS of a wheat–legume mixture (e.g., 70:30 ratio) can approach 0.8–0.9, approaching the quality of animal proteins [55].

Several strategies can improve the amino acid profile and protein quality of wheat-based foods: (1) blending wheat flour with legume flours (soy, chickpea, lentil); (2) germination and sprouting, which increases lysine content through proteolytic activity; (3) fermentation, which can increase free amino acid content; and (4) genetic improvement of wheat varieties with enhanced lysine content [46,53].

### 3.2. Carbohydrate Composition

Wheat is primarily a carbohydrate source, with starch constituting approximately 60–75% of the grain weight. Starch exists in two forms: amylose (linear α-1,4-glucan, 20–30%) and amylopectin (branched α-1,4 and α-1,6-glucan, 70–80%). The amylose-to-amylopectin ratio significantly affects glycemic response, with higher amylose content associated with lower postprandial glycemic index [53].

Wheat also contains non-starch polysaccharides (NSPs), including arabinoxylans (5–8% of grain weight), β-glucans (0.5–2%), and cellulose (2–3%). Arabinoxylans are the predominant dietary fiber component in wheat bran and are composed of a xylan backbone with arabinose substitutions. These NSPs resist digestion in the upper gastrointestinal tract and are fermented by colonic microbiota, producing short-chain fatty acids (SCFAs) including acetate, propionate, and butyrate [27,56]. The soluble fiber fraction (primarily arabinoxylans and β-glucans) contributes to viscosity modulation and cholesterol-lowering effects, while insoluble fiber (cellulose, lignin) promotes stool bulk and regularity [53].

### 3.3. Lipid Composition

Wheat lipids constitute approximately 1.5–3.0% of the grain weight, with the germ fraction containing the highest lipid concentration (8–12% of germ weight) [53]. The fatty acid profile of wheat lipids is dominated by polyunsaturated fatty acids (PUFAs), particularly linoleic acid (C18:2, 50–60% of total fatty acids), followed by oleic acid (C18:1, 15–25%) and palmitic acid (C16:0, 15–20%) [53]. The germ is also rich in tocopherols and tocotrienols (collectively vitamin E), with α-tocopherol as the predominant form (approximately 60% of total vitamin E). These lipid-soluble antioxidants contribute to the oxidative stability of wheat germ oil and provide cardioprotective effects [48,53].

### 3.4. Mineral Composition and Bioavailability

Wheat is a significant source of essential minerals, though their concentration and bioavailability vary considerably across grain fractions. The bran and aleurone layers contain the highest mineral concentrations, while the endosperm contains substantially lower levels (Table 2).

The bioavailability of minerals from wheat is significantly influenced by the presence of phytic acid (myo-inositol hexaphosphate), which is concentrated in the bran and aleurone layers. Phytic acid forms insoluble complexes with divalent cations (Fe^2+^, Zn^2+^, Ca^2+^, Mg^2+^), reducing their intestinal absorption [53]. This explains why iron and zinc from whole wheat are less bioavailable than from animal sources. Several strategies can enhance mineral bioavailability from wheat:

(a) Phytase activation through germination or fermentation, which hydrolyzes phytic acid and releases bound minerals [27].
(b) Sourdough fermentation, where microbial phytases reduce phytate content by 40–60% [57]
(c) Fortification of wheat flour with mineral salts
(d) Genetic selection for low-phytate wheat varieties

The practical implication is that although whole wheat contains higher mineral concentrations, the bioavailability of certain minerals (particularly iron and zinc) may be lower than refined flour due to phytate content. However, the overall nutritional benefits of whole wheat (higher fiber, phenolics, and other bioactive compounds) generally outweigh this limitation, and processing strategies can mitigate phytate effects [48,53].

### 3.5. Vitamin Composition

Wheat is a rich source of B-complex vitamins, with the bran and germ fractions containing the highest concentrations. Thiamine (vitamin B1), riboflavin (vitamin B2), niacin (vitamin B3), pyridoxine (vitamin B6), and folate (vitamin B9) are present in significant amounts. The germ is particularly rich in vitamin E (tocopherols and tocotrienols) and contains small amounts of vitamin K [48,53] (Table 2).

The B-vitamin content of wheat is particularly important for energy metabolism, as these vitamins serve as cofactors for enzymes involved in carbohydrates, fat, and protein metabolism. Thiamine is essential for pyruvate dehydrogenase and α-ketoglutarate dehydrogenase, riboflavin for flavin adenine dinucleotide (FAD)-dependent enzymes, niacin for nicotinamide adenine dinucleotide (NAD)-dependent dehydrogenases, and pyridoxine for amino acid metabolism [53]. Folate is critical for one-carbon metabolism and DNA synthesis, with particular importance during pregnancy.

The substantial loss of vitamins during milling (refining) is a key nutritional argument for whole-grain consumption. White flour typically contains 60–80% less B vitamins and 90–95% less vitamin E than whole grain [53]. This has led to mandatory fortification of refined wheat flour in many countries (e.g., folic acid fortification in the United States and Canada) to prevent deficiency-related conditions such as neural tube defects [48].

## 4. Nutraceutical and Health Benefits of Wheat

### 4.1. Antioxidant Activity

Wheat-derived phenolic compounds reduce oxidative stress by scavenging reactive oxygen species (ROS). This activity has been demonstrated in chemical assays and cell-based models; however, chemical antioxidant capacity (DPPH, ABTS, FRAP) should be distinguished from biological antioxidant effects in vivo. The evidence for systemic antioxidant effects in humans is currently limited and requires further investigation. Antioxidant activity varies among wheat varieties. Red wheat flour shows stronger DPPH radical-scavenging activity, with a half-maximal inhibitory concentration (IC50) of 121.68 μg/mL, compared to durum wheat (IC50 198.34 μg/mL) and soft wheat flour (IC50 226.70 μg/mL) [58]. All values have been standardized to μg/mL for consistency.

Antioxidant activity is closely associated with phytochemical composition. Compounds such as chlorogenic acid, caffeic acid, luteolin, and apigenin contribute to redox balance, particularly under environmental stress [59]. Additionally, a polysaccharide from Agrocybe aegerita-fermented wheat aleurone reduces ROS, lipid peroxidation, and oxidative damage. This compound also increases antioxidant enzyme activities, including superoxide dismutase (SOD), glutathione peroxidase (GSH-Px), and catalase [60].

Processing methods, such as germination and fermentation, can enhance wheat bioactivity. Ultrasound-assisted germination of wheat increases gamma-aminobutyric acid levels to 18 mg/100 g and flavonoid content to 0.19 mg QE/g. This results in a greater antioxidant capacity of 2.86 mg/g [61]. Wheat bran polysaccharides and fermented derivatives also show strong free radical scavenging activity. They protect against oxidative damage by inhibiting lipid peroxidation and reducing intracellular ROS levels [43,44].

Recent studies highlight the protective effects of wheat-derived bioactive peptides. Peptides isolated from wheat germ protein (YFGWPGPK, FGWPGPR, and GHHWPLPP) protect H2O2-damaged PC12 cells. These peptides reduce ROS and malondialdehyde (MDA) levels and increase antioxidant enzyme activity [62]. In vitro assays, including 2,2′-azino-bis(3-ethylbenzothiazoline-6-sulfonic acid) (ABTS), DPPH, ferric reducing antioxidant power (FRAP), and hydroxyl radical scavenging, support the antioxidant potential of wheat-derived peptides [63]. Structurally modified wheat bran peptides show enhanced bioactivity with improved free radical scavenging and functional properties [64]. In vitro studies show that black wheat bran treated with steam explosion at 1.0 MPa for 90 s has the highest levels of soluble fiber, phenolics, flavonoids, and anthocyanins. This treatment enhances antioxidant activity, with DPPH scavenging of 37.5%, ABTS scavenging of 31.83%, and FRAP of 45.82% [65].

Wheat product extracts enhance free radical scavenging capacity and stimulate immune responses by modulating key immune markers [66]. Recent findings show that colored wheat bran varieties (white, red, green, blue, purple, and black) contain 90 bioactive phytochemical constituents, with ferulic acid being particularly abundant. Advanced high-resolution liquid chromatography–mass spectrometry analysis shows a strong correlation between phenolic composition and antioxidant activity [22]. Food fortification and enzymatic modification further improve antioxidant properties. Ficin hydrolysis of wheat gluten improves dough properties and texture while enhancing nutritional functionality. Hydrolysis releases bioactive peptides and modified wheat gluten with notable antioxidant activity, supporting wheat-based functional food development [67,68].

The primary antioxidant mechanism of wheat phenolic compounds involves scavenging ROS and chelating transition metal ions, protecting cellular components from oxidative damage in vitro [41]. However, these chemical and cell-based findings do not establish that wheat consumption produces clinically meaningful systemic antioxidant effects or reduces disease risk in humans. Table 3 summarizes the nutritional and antioxidant properties of wheat varieties and derivatives, with explicit indication of evidence levels.

### 4.2. Diabetes Management Using Wheat

DM is a serious metabolic disorder characterized by persistent hyperglycemia caused by β-cell dysfunction or impaired insulin action. The International Diabetes Federation (IDF) data shows that 537 million adults had diabetes globally in 2021. This number may rise to 643 million by 2035 [70]. Many whole-grain-derived phytochemicals, including wheat-derived compounds, may attenuate DM effects and have shown prevention potential. Epidemiological and clinical evidence indicate that whole-meal wheat flour products with improved glycemic control and reduced T2D risk [71,72], largely due to their high fiber content and bioactive compounds. A cohort analysis of 160 participants with diabetes showed that wheat consumption (40 g/day for 30 days) is associated with DM management [73]. However, these observational findings do not establish causation, and the effect size of wheat consumption on glycemic outcomes in humans requires confirmation through randomized controlled trials (RCTs).

An in vitro study demonstrated that wheat bran soluble dietary fiber inhibits α-amylase and α-glucosidase [74]. Yogurts fortified with purple wheat flour exhibit significant α-glucosidase inhibitory activity and a rich bioactive compound profile [75]. Total phenolics and anthocyanins enhance antioxidant capacity and improve glycemic control. Similarly, anthocyanin-rich black wheat chapatti shows stronger α-amylase and α-glucosidase inhibitory activity than conventional white wheat chapatti, improving glycemic control [76]. Protein and phytic acid contents in wheat bran significantly lower glycemic index values (*p* ≤ 0.05) by limiting starch gelatinization and enzyme accessibility [77]. Bioactive peptides derived from soft and hard wheat gluten exhibit α-amylase (53.3%) and α-glucosidase (18.4%) inhibitory activities [74]. These enzyme inhibition assays are hypothesis-generating and do not establish clinical efficacy.

Many studies have evaluated the effects of wheat bioactive peptide products on glucose metabolism and diabetes-related outcomes. Bioactive peptides improve glucose metabolism and insulin signaling pathways [78] and suppress glucose transport after digestion [79]. Peptides derived from soft and hard wheat gluten exhibit the highest α-amylase (53.3%) and α-glucosidase (18.4%) inhibitory activities [74]. In animal models, wheat-derived non-starch polysaccharides significantly improve blood glucose regulation, lipid profiles, and hepatic and renal functions in streptozotocin (STZ)-induced diabetic C57BL/6J mice [80].

An anthocyanin-rich fraction from the purple wheat cultivar Ari-heukchal (200 µg/mL) significantly increases AKT phosphorylation at Ser473 and restores glucose uptake in HepG2 cells [81]. Anthocyanin-rich black wheat chapatti significantly reduces blood glucose and hemoglobin A1c levels while enhancing insulin sensitivity and glucose tolerance in high-fat diet and STZ-induced T2DM rats [76]. Mechanistically, these effects are mediated through activation of AMPK and PI3K/AKT pathways (as detailed in Section 4.8.3 and Section 4.8.4), upregulating glucose transporter (GLUT) 2 and GLUT4 expression [76]. High-fiber whole wheat noodle diets significantly upregulate insulin receptor (IR), IR substrate 2 (IRS-2), GLUT2, and AKT, activating PI3K and enhancing tissue glucose uptake, thereby reducing blood glucose levels in T2DM rats [82]. Wheat germ peptide activates and promotes glycogen synthase kinase 3 beta phosphorylation and increases forkhead box protein O1 and GLUT2 expression. Together, these effects enhance glycogen synthesis, inhibit gluconeogenesis, and promote glucose transport in insulin-resistant HepG2 cells [83]. Bread made from 100% wheat flour suggests beneficial effects on glucose regulation in individuals with T2D [84]. This study identified wheat flour as a dietary factor influencing energy homeostasis and body weight regulation [85].

More recently, sprouted wheat exerts protective effects by reshaping gut microbiota composition, suppressing the toll-like receptor 4 (TLR4)/nuclear factor kappa B inflammatory pathway, and regulating five key bioactive metabolites. These metabolites included 2α-hydroxyursolic acid, apigenin, epigallocatechin, naringenin, and gallocatechin. Four core gene targets were identified: PIK3R1, AKT1, TLR4, and tumor necrosis factor alpha (TNF-α) [86].

Model-Specific Limitations: The STZ-induced diabetic model involves chemical destruction of pancreatic β-cells and does not fully recapitulate the pathophysiology of human T2D, which typically involves insulin resistance with progressive β-cell dysfunction. HFD-induced obesity models better represent insulin resistance but do not capture the full metabolic complexity of human diabetes. Combined HFD/STZ models induce both insulin resistance and β-cell damage but remain imperfect representations of human disease. Findings from these models should be considered hypothesis-generating rather than evidence of clinical efficacy.

**Figure 1:**
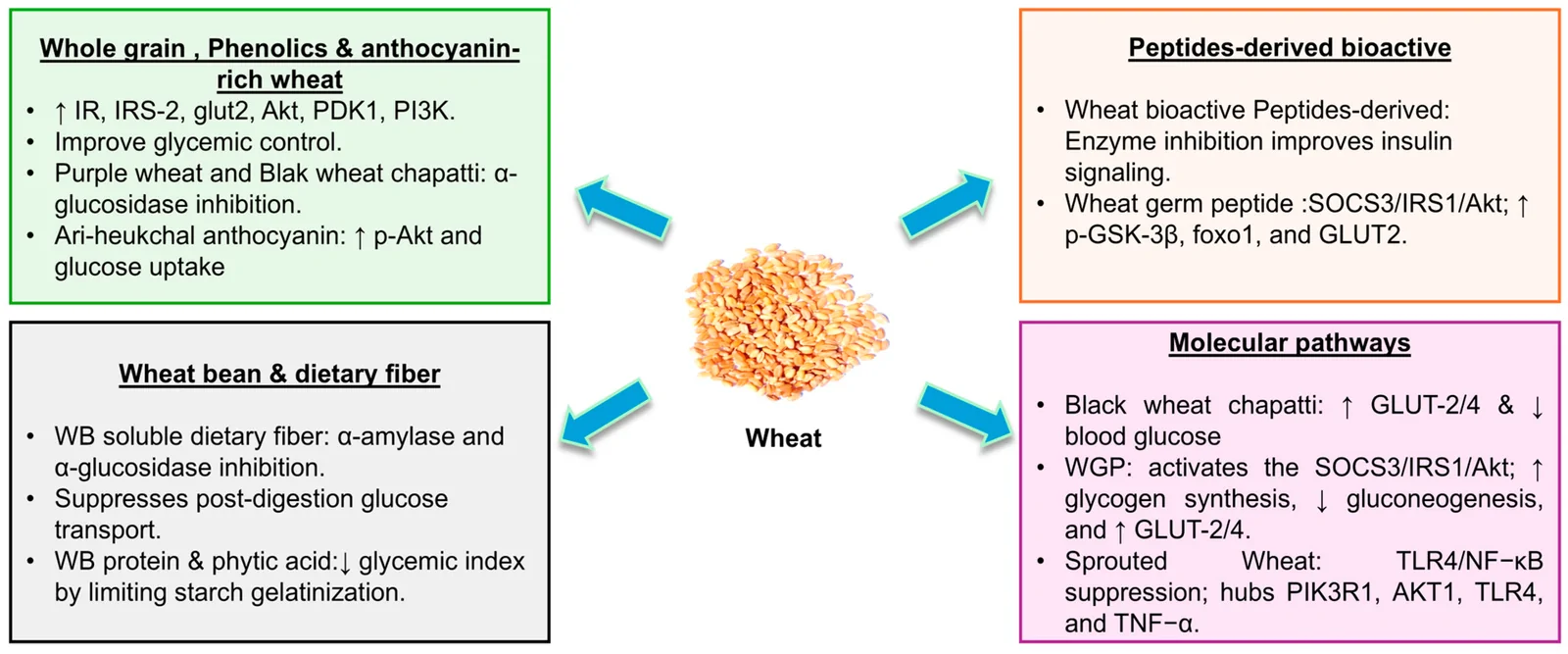
Antidiabetic mechanisms of wheat-derived phytochemicals, peptides, and dietary components. WB, wheat bran; SW, sprouted wheat; WGP, wheat germ peptide; GLUT, glucose transporter; HFD, high-fat diet; STZ, streptozotocin; IRS-2, insulin receptor substrate; TLR4, toll-like receptor 4; NF-κB, nuclear factor kappa B; TNF-α, tumor necrosis factor alpha; SOCS3, suppressor of cytokine signaling 3; GSK-3β, glycogen synthase kinase-3 beta; FOXO1, forkhead box protein O1. ↑ increase and ↓ decrease.

Overall, wheat-derived compounds exhibit antidiabetic activity in preclinical models, reducing serum blood glucose levels through multiple mechanisms including enzyme inhibition, enhanced insulin signaling, and gut microbiota modulation. However, clinical translation requires rigorous human RCTs to establish definitive efficacy and safety. Figure 1 and Table 4 summarize the antidiabetic effects and mechanisms of wheat-derived compounds in preclinical models, with explicit evidence levels.

### 4.3. Obesity Management Using Wheat-Derived Bioactive Components

Obesity is a progressive chronic metabolic disorder and a major global public health challenge. Its global prevalence is rising rapidly. The Global Burden of Disease 2021 study projects that, if current trends continue, more than 3.8 billion adults will be overweight or obese by 2050 [87]. This would represent over half of the global adult population, with the fastest increases expected in low- and middle-income countries. This trend has persisted for decades despite increased awareness of associated health risks and prevention strategies.

Although wheat is recognized for its functional food benefits, certain components may have adverse effects under specific conditions. For instance, oral wheat gliadin challenge induces enteropathy by inhibiting arachidonic acid metabolism, an omega-6 polyunsaturated fatty acid pathway. This highlights the complex dual role of wheat components in metabolic health [88].

Emerging evidence indicates that wheat derivatives contribute to obesity management and modulate gut microbiota. In vitro results confirm that wheat beta-glucan significantly reduces obesity-associated bacterial genera, including Coriobacteriaceae UCG-002, Romboutsia, Faecalibaculum, and Enterorhabdus, thereby improving metabolic outcomes [89]. Similarly, wheat arabinoxylans exert prebiotic effects by enhancing short-chain fatty acid (SCFA) production, particularly butyrate. They also improve gut barrier integrity and suppress inflammatory mediators, including TNF-α and interleukin-6 (IL-6) [56]. In obese mice, 5% wheat bran alone was ineffective, whereas oral CbXyn10C administration reduced body weight and lipid accumulation, improved glycolipid metabolism, reduced systemic inflammation, and strengthened intestinal barrier function in HFD-induced obesity [90].

The lipid-rich fraction of wheat also significantly promotes metabolic regulation. Wheat bran treatment reduces body and liver weights, increases SOD and GSH-Px activities, decreases MDA content, and increases GLP-1 secretion [91]. It also ameliorates hepatic steatosis and inflammation in the HFD model through gut microbiota modulation and lipid metabolism regulation [92]. Fermented wheat germ is more effective than unfermented wheat germ in reducing body weight gain and fat accumulation, improving glycolipid metabolism, and alleviating inflammation [93]. Furthermore, wheat germ and fermented wheat germ supplementation are associated with improved placental function, reduced lipid accumulation, lower inflammatory marker levels, and reduced oxidative stress [94].

At the molecular level, wheat alkylresorcinols reduce SIRT1 expression following miR-34a mimic transfection. This is accompanied by inhibited slow-twitch fiber formation, myogenesis, and differentiation. Conversely, alkylresorcinol pretreatment reversed this effect, indicating that alkylresorcinols promote slow-twitch fiber formation, myogenesis, and differentiation via the miR-34a/SIRT1 axis [95].

Wheat peptides exert multi-target effects against HFD-induced metabolic disturbances by suppressing inflammation, restoring lipid metabolism, and modulating gut microbiota. They potently inhibit dipeptidyl peptidase IV (DPP-IV) via the tripeptide leucine–proline–glutamine and tetrapeptide leucine–proline–glutamine–phenylalanine [96]. In addition, hydroxytyrosol-enriched wheat bread reduces body fat mass and improves fasting glucose, insulin, and HbA1c levels [97].

Anthocyanin-biofortified colored wheat (purple, blue, and black) has also emerged as a promising functional cereal for metabolic health. In HFD mice, black and purple wheat reduced serum total cholesterol, triglycerides, and free fatty acids. They also improved blood glucose regulation and insulin sensitivity. Black wheat additionally reduced body weight gain and adipose tissue mass by upregulating fatty acid β-oxidation genes (including crat, acca2, and lonp2) and oxidative stress response genes [38]. An earlier review supported including colored wheat anthocyanins in obesity prevention strategies and discussed their broader health effects [1]. Table S3 provides a comparative summary of in vivo efficacy of different wheat fractions (germ, bran, whole grain) and colored wheat varieties (purple, blue, black) in animal models of metabolic disease, highlighting the superior effects of fermented wheat germ and anthocyanin-rich black wheat.

Extracts from Korean wheat cultivars “Shinmichal” and “Saekeumkang” significantly reduce hepatic lipid accumulation, total cholesterol, and triglycerides while improving cardiovascular risk markers in HFD-fed mice [98]. A wheat aleurone-enriched diet increased postprandial butyrate production and reduced oxidative stress markers in individuals with cardiometabolic risk [99].

In HFD-fed rats, lipophilic wheat extracts (200 mg/day) reduce body weight gain, improve lipid profiles, reduce inflammation, and inhibit adipogenesis by activating the Wnt/beta-catenin pathway [100]. Insoluble dietary fiber from wheat bran reduces lipid levels and regulates intestinal microbiota in animal models [101]. Sprouted wheat modulates gut microbiota composition and inhibits the TLR4/NF-κB inflammatory pathway in mouse models [86]. The predominance of bound phenolics in wheat bran further highlights the importance of colonic fermentation in mediating their bioactivity [27].

Human Evidence: Functional wheat-based foods show promising effects in clinical settings. Wheat biscuits enriched with plant proteins contributed to greater weight loss, lower energy intake, and reduced insulin resistance in a 12-week RCT involving overweight and obese individuals [102]. Hydroxytyrosol-enriched wheat bread reduced body fat mass and improved fasting glucose, insulin, and HbA1c levels in patients with overweight/obesity and T2D [97]. A randomized controlled trial in adults with obesity showed that whole-grain consumption favorably affects body weight and cardiometabolic risk factors [97]. However, a meta-analysis of 21 RCTs reports that whole-grain consumption does not consistently affect anthropometric measures including body weight, BMI, waist circumference, or fat mass [103]. This apparent tension suggests that whole-grain benefits may be mediated through mechanisms beyond simple weight loss (e.g., improved insulin sensitivity, reduced inflammation) and may depend on the specific wheat fraction, baseline population characteristics, and study duration.

Dose Considerations: Animal doses in obesity studies often exceed achievable human intake. For example, wheat germ extract doses of 200 mg/kg/day in rats translate to approximately 1200 mg/day for a 60 kg human when adjusted for body surface area, which may not be achievable through dietary wheat consumption. This limitation should be considered when interpreting preclinical findings.

**Figure 2:**
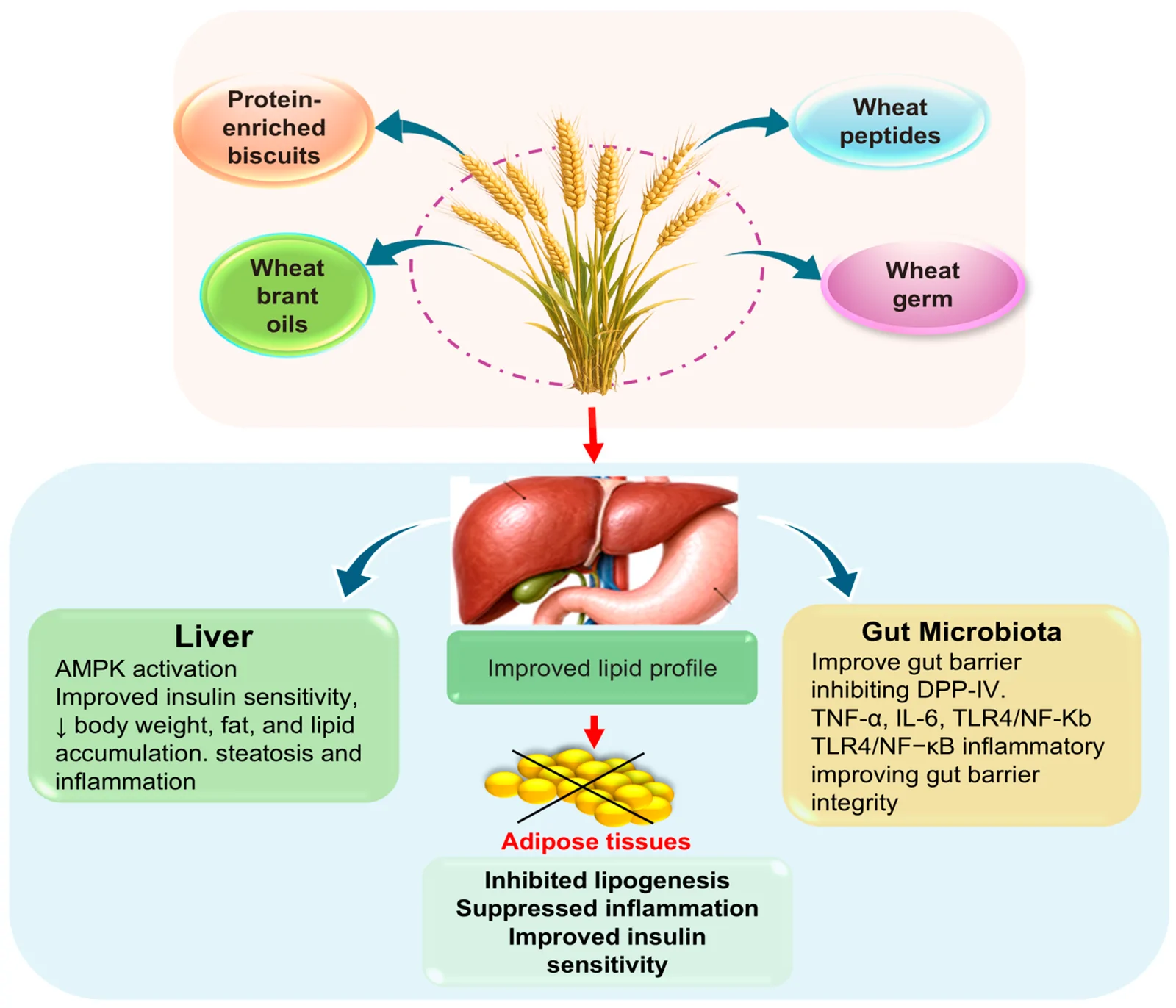
Wheat-derived components and their mechanism of action in metabolic regulation and obesity management. Wheat-derived bioactive compounds may improve metabolic health by regulating liver lipid metabolism, adipose tissue lipogenesis, inflammation, and insulin sensitivity. They also modulate gut microbiota, contributing to overall anti-obesity effects. ↓ decrease.

Figure 2 and Table 5 summarize the anti-obesity effects of wheat-derived compounds in preclinical models. These findings support the development of wheat-based functional foods, including germ, sprouted wheat, colored wheat varieties, and fermented wheat products, as promising dietary strategies for obesity management. Clinical translation requires larger, longer-term RCTs with standardized interventions and outcomes.

### 4.4. Anti-Inflammatory Effects of Wheat-Derived Bioactive Compounds

Inflammation is a protective biological response that defends the host against pathogens and maintains tissue homeostasis [104]. Macrophages are crucial for inflammation by producing pro-inflammatory mediators, such as nitric oxide, chemokines, and cytokines [105]. Cumulative evidence indicates that wheat-derived bioactive compounds exhibit significant anti-inflammatory properties through multiple mechanisms, primarily involving inhibition of NF-κB and MAPK inflammatory pathways (Section 4.8.2 and Section 4.8.5).

Wheat phytochemicals modulate inflammatory pathways by inhibiting pro-inflammatory cytokine production and related signaling cascades in cell and animal models. Hulls from gamma-irradiated mutant lines of *Triticum aestivum* exhibit altered phytochemical profiles and enhanced anti-inflammatory activity in macrophage assays [106]. Fermented wheat products from Algeria showed anti-inflammatory activity in vitro, with inhibition rates ranging from 20% to 39% [107].

Ferulic acid, a predominant phenolic acid in wheat, has been identified in multiple studies as an anti-inflammatory and immunomodulatory compound in preclinical models. A ferulic acid-rich wheat bran ingredient reduced TNF-α, IL-6, and IL-1β production in a murine model of graft-versus-host disease [108]. Wheat bran polyphenols ameliorated dextran sulfate sodium (DSS)-induced ulcerative colitis in mice by suppressing mitogen-activated protein kinase (MAPK) and NF-κB inflammasome pathways [109]. These effects are accompanied by changes in the intestinal microbiota, supporting a mechanistic link between wheat polyphenols, gut microbiota, and inflammation in animal models [102,109].

Wheat seedling extracts administered at 200 mg/kg and 400 mg/kg significantly reduced pro-inflammatory factors including TNF-α, IL-6, and IL-1β in aged mice. The extracts also improved mitochondrial function by activating the phosphorylated AMPK, sirtuin 3 (SIRT3), and peroxisome proliferator-activated receptor gamma coactivator 1-alpha pathway [110].

Extracts of Xianju wheat pastes inhibited nitric oxide production in macrophage assays. The ethyl acetate extract showed the strongest inhibitory effect with an EC50 of 61.92 μg/mL and suppressed TNF-α, IL-6, and IL-1β production at 40 μg/mL [111]. Wheat bran modulated gut microbiota composition in DSS-induced inflammation models and increased the abundance of beneficial bacterial groups including Escherichia, Allobaculum, and Bacteroidaceae [112]. Anthocyanins in colored wheat, particularly cyanidin, delphinidin, and pelargonidin glucosides, exert anti-inflammatory effects by inhibiting NF-κB signaling and modulating pro-inflammatory cytokine production in cell models [20].

Wheat germ-derived products exhibit anti-inflammatory and antioxidant effects in animal models. Wheat germ oil reduced oxidative stress, suppressed inflammation and NF-κB signaling, and decreased kidney injury molecule 1 (KIM-1) expression. It also activated the nuclear factor erythroid 2-related factor 2 (Nrf2) and heme oxygenase 1 (HO-1) antioxidant pathway in ethanol-induced hepatorenal injuries in rats [113]. Wheat germ oil, containing high levels of linoleic acid (45.3%), squalene (2.52 g/100 g), and polyphenols, reduced lipopolysaccharide (LPS)-induced nitric oxide and IL-6 production in macrophages [114]. Low-dose wheat germ increased total antioxidant capacity and reduced serum markers including MDA, LPS, and diamine oxidase in animal models, indicating improved intestinal barrier function [115].

Wheat peptides protect intestinal epithelial cells in vitro. Pretreatment with wheat peptides reduced LPS-induced permeability in Caco-2 cells, inhibited activation of the NF-κB p65 and MAPK signaling pathways and reduced inflammatory factor expression. Wheat peptides also increased tight junction protein expression, improving intestinal barrier functions [116]. Arriheuk wheat sprout extract mitigated dexamethasone-induced muscle atrophy in C2C12 cells and mouse models by activating the AKT/mammalian target of rapamycin (mTOR) pathway and the AMPK/forkhead box O3 (FOXO3) signaling pathways, while suppressing atrogin-1, muscle RING finger protein 1, and myostatin expression [117].

Fermented wheat germ extract (FWGE) is widely investigated for its anti-inflammatory and antioxidant potential. Treatment with FWGE (1–2%) significantly reduced intracellular ROS levels in LPS-stimulated cells [118]. FWGE reduced ROS production and lipid peroxidation in in vitro models [119]. A recent systematic review synthesized evidence that FWGE improves inflammatory and immune parameters across models of cancer, autoimmune disease, and metabolic disease [120].

FWGE, bioprocessing strategies including enzymatic hydrolysis, hydrothermal treatment, and high-pressure processing can release bound ferulic acid and feruloyl arabinoxylan oligosaccharides from wheat bran, generating soluble ingredients with enhanced antioxidant and anti-inflammatory activity in LPS-stimulated macrophages [49,50].

Evidence Level Summary: The anti-inflammatory effects of wheat-derived compounds are supported by Level 3 (animal studies) and Level 4 (cell-based experiments) evidence. Limited human data are available; the clinical translation of these findings requires further investigation through well-designed RCTs with inflammatory biomarkers as primary or secondary endpoints. Table 6 summarizes the anti-inflammatory effects of wheat-derived compounds in preclinical models.

### 4.5. Anticancer Potential of Wheat-Derived Phytochemicals

Globally, approximately 20 million new cancer cases are diagnosed every year, and approximately one in five people develops cancer during their lifetime [50]. Wheat-derived phytochemicals have attracted attention for their antiproliferative activities in preclinical models because they can induce apoptosis and disrupt tumor growth-related pathways in vitro. However, these findings support mechanistic hypotheses that warrant further investigation; they do not establish clinical efficacy for cancer prevention or treatment. The anticancer section is dominated by cultured cancer cell studies, purified compounds, and animal models. Evidence-calibrated terminology (cytotoxic, cytostatic, pro-apoptotic, antiproliferative) is used throughout.

Fermented Wheat Germ Extract (FWGE): In cancer cell models, FWGE significantly inhibits the migration and invasion of HSC-3 and SCC-25 cells at 10 mg/mL, while SAS cells show inhibition only at 10 mg/mL [121]. FWGE is evaluated as both a monotherapy and a combination treatment with chemotherapeutic agents. In rat cancer models, oral FWGE combined with 5-fluorouracil inhibits liver metastases [122]. FWGE demonstrates antiproliferative activity against non-Hodgkin lymphoma and inhibits the proliferation of non-small cell lung cancer cells in both in vitro and in vivo models [123]. FWGE exerts antiproliferative effects by disrupting tumor cell metabolism and inducing apoptosis [120].

FWGE (10 mg/mL) shows cytotoxic responses in pancreatic cancer cells (ASPC-1 and BxPC3) and breast cancer cells (MDA-MB-231 and MA-MB-468), with cytostatic effects observed in gastric, breast, and colon cancer models. FWGE also increased LC3-II levels (a marker of autophagy) and reduced glucose uptake and lactate production, indicating metabolic reprogramming [124]. The bioactive components of FWGE include the quinones 2-methoxy-1,4-benzoquinone and 2,6-dimethoxy-1,4-benzoquinone [120]. FWGE suppresses the Warburg effect and restores oxidative mitochondrial metabolism in tumor cells [125].

Other Wheat Fractions: Blue wheat bran extracts show no significant effect on colorectal cancer (CRC) cell viability, whereas purple wheat bran extract protects against oxidative stress under exposure to 50 µM H2O2 in cell models [126]. Similarly, purple- and blue wheat bread-derived extracts show stronger inhibitory activity against HCT-15 cells than red or black wheat extracts, suggesting higher phenolic bioavailability in these wheat varieties [127].

A recent molecular study shows that wheat extract suppresses proliferation of the A549 lung cancer cell line, potentially by altering ROS homeostasis, with greater activity observed in irradiated grains [128].

Ferulic Acid: Ferulic acid inhibits the proliferation and migration of colon cancer cells across different Duke stages by activating the ataxia telangiectasia mutated/checkpoint kinase 2 (Chk2) pathway and ataxia telangiectasia and Rad3-related (ATR)/Chk1 pathway. Ferulic acid downregulates CDK2 and cyclin A2, along with CDK4, CDK6, cyclin D1, and cyclin E1 complexes, leading to S-phase arrest in SW-480 and Caco-2 cells and G1-phase arrest in HCT-116 cells. It also upregulates p53 and p21 [128].

A recent comprehensive review of the anticancer activity of ferulic acid in breast, colon, prostate, and cervical cancers reports multiple mechanisms, including inhibition of PI3K, AKT, mTOR, Notch, and Wnt signaling; reduction in vascular endothelial growth factor and matrix metalloproteinase-9; and induction of apoptosis, primarily associated with reduced epidermal growth factor receptor expression [129].

Wheat Bran and Polysaccharides: Wheat bran hydrolysates are associated with anticancer and immunomodulatory effects. Treatment with wheat bran hydrolysate at 0.5 mg/mL increased the production of pro-inflammatory cytokines, including TNF-α, IL-6, and IL-1β, and reduced CRC- and macrophage-mediated mechanisms in RAW264.7 cells [130]. Furthermore, wheat cell culture polysaccharides (WCCPSs), comprising six fractions, induced apoptosis in HCT-116 cells via both intrinsic and extrinsic pathways, increasing Bcl-2-associated X protein (Bax), caspase-8, caspase-3, and cytochrome c [131]. Subsequent studies show that WCCPS also suppresses β-catenin signaling and cancer cell markers, suggesting potential roles in differentiation and apoptosis in HCT-116 CRC cells [132].

Wheat bran antioxidant extracts at 100 μg/mL, 500 μg/mL, and 1000 μg/mL inhibit the growth of SW480 colon cancer cells via oxidative stress-induced apoptosis. These extracts promote HepG2 cell growth but show minimal effects on HeLa cervical cancer cells. Evidence from rat models also supports the chemopreventive potential of wheat components [11].

Animal Studies: A diet supplemented with wheat germ powder or wheat germ oil (270 mg/kg body weight/day) reduces tumor size and improves biochemical parameters in a 7,12-dimethylbenz[a]anthracene-induced carcinogenesis model [133]. Additionally, wheat germ oil demonstrates protective effects against tumorigenesis in infected immunosuppressed hosts [134]. At the molecular level, wheat germ oil at 7.5 μg/mL and 15 μg/mL increases apoptosis and necrosis, downregulates BCL-2 expression (*p* < 0.05), and upregulates P53 and BAX expression in MCF-7 and HeLa cancer cell lines [135].

Delivery Systems and Clinical Evidence: Novel delivery systems also show promise to increase the efficacy of chemotherapeutic agents containing wheat-derived components. Wheat germ agglutinin-conjugated chitosan nanoparticles are used for improving the targeted delivery of cytotoxic agents [136]. FWGE has advanced to clinical trials, demonstrating supportive benefits as an adjunct therapy for castration-resistant prostate cancer [137]. However, current clinical evidence remains limited by methodological heterogeneity, small sample sizes, and the absence of placebo-controlled trials.

**Figure 3:**
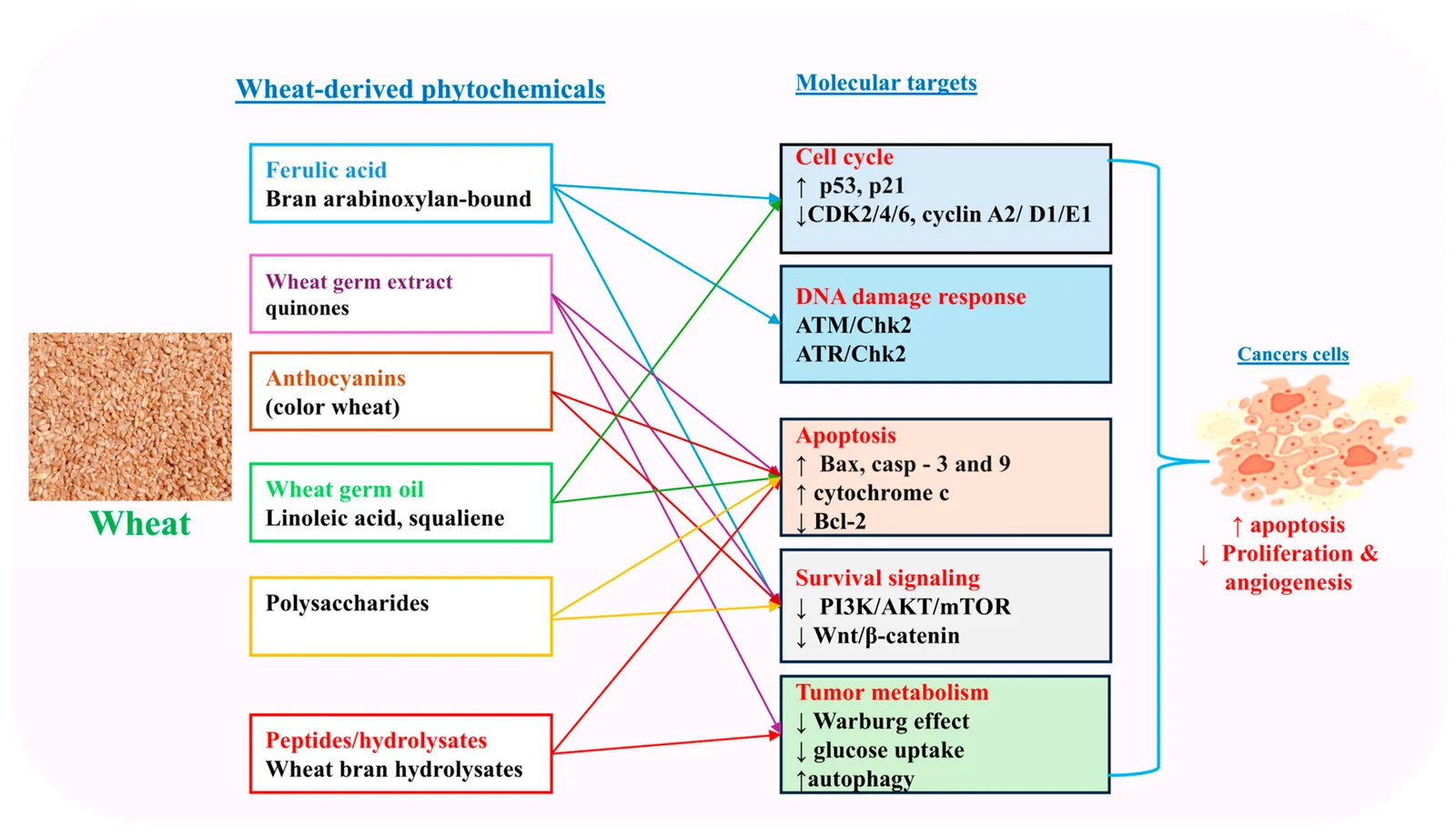
**Molecular targets of wheat-derived phytochemicals in cancer.** This figure illustrates the cellular pathways modulated by wheat-derived compounds (ferulic acid, FWGE, anthocyanins, alkylresorcinols, and bioactive peptides) in cancer cell models. Evidence level indicators: Solid arrows = experimentally demonstrated (Level 4: cell-based); dashed arrows = inferred or hypothesized (Level 6: computational predictions); green boxes = validated targets with multiple studies; yellow boxes = emerging targets requiring further validation. Abbreviations: AKT, protein kinase B; ATM, ataxia telangiectasia mutated; CDK2, cyclin-dependent kinase 2; Chk1/2, checkpoint kinases 1 and 2; mTOR, mechanistic target of rapamycin; PI3K, phosphoinositide 3-kinase. ↑ increase ↓ decrease.

Evidence Level Summary: The anticancer effects of wheat-derived compounds are supported by Level 3 (animal studies) and Level 4 (cell-based experiments) evidence, with limited Level 2 (observational human studies) and Level 1 (RCT) data. The clinical significance of these findings remains to be established. Figure 3 summarizes the molecular targets of wheat-derived phytochemicals in cancer, with evidence levels clearly indicated.

### 4.6. Antimicrobial Activities of Wheat

Whole-meal flours show antimicrobial activity against both Gram-negative and -positive bacteria, promoting the growth of *Lactobacillus brevis*, a potential probiotic bacterium [15]. The green extraction and quantification of azelaic acid from whole grains and whole-grain flour of *T. durum* Desf. Pure azelaic acid at 256 μg/mL shows no antibacterial activity against *Staphylococcus aureus*, *Escherichia coli*, or *Pseudomonas aeruginosa* but exhibits a minimum inhibitory concentration of 256 μg/mL against *Streptococcus pyogenes* [138].

Anthocyanin-biofortified colored wheat varieties, including purple, blue, and black wheat, have emerged as valuable sources of antimicrobial compounds. Sharma et al. report that black, purple, and blue wheat flour and wheatgrass juice at 200 mg/mL exhibit strong antibacterial and antifungal activity against common human pathogens, including *Staphylococcus aureus*, *Escherichia coli*, *Candida albicans*, and *Pseudomonas aeruginosa*. The major anthocyanins identified include cyanidin-3-O-glucoside chloride, delphinidin-3-O-galactoside, and delphinidin-3-O-glucoside [38].

Recent reviews confirm that colored wheat anthocyanins, particularly cyanidin, delphinidin, and pelargonidin glucosides, exert antibacterial effects through membrane destabilization, microbial enzyme inhibition, and disruption of biofilm formation [20]. Pure compounds and fermentation-, germination-, or enzymatic hydrolysis-induced bioprocessed wheat ingredients improve the release of bound phenolics and generate novel bioactive peptides with greater antimicrobial activity [27,139].

### 4.7. Wheat-Mediated Modulation of the Gut Microbiome

Whole-wheat fiber promotes the growth of beneficial gut microbiota, supporting digestive health and immune regulation. Wheat bran dietary fiber may confer metabolic benefits by inhibiting the 1,2-diacylglycerol (DAG)/protein kinase C epsilon (PKCε) pathway, thereby improving insulin receptor activity and insulin signaling. High-throughput 16S rDNA sequencing shows that wheat bran dietary fibers significantly increase the relative abundance of *Akkermansia muciniphila*, enhance overall microbial diversity, and reduce the Firmicutes-to-Bacteroidetes ratio [140]. However, the Firmicutes-to-Bacteroidetes ratio is not a universal marker of improved metabolic health; findings vary across studies, and absolute abundance, functional capacity, and SCFA production are more informative.

Mechanistically, wheat-derived arabinoxylan modulates gut microbiota composition, promotes colonic regulatory T-cell expansion, and enhances mucosal immunoglobulin A (IgA) responses, thereby strengthening gut barrier integrity and immune function [141]. Wheat germ protein-derived peptides, including VPIPNPSGDR, VPY, and AR, obtained from defatted wheat germ protein hydrolysates, exhibit multiple biological activities, including anti-adhesive activity against *Helicobacter pylori* and inhibitory activity against angiotensin-converting enzyme and DPP-IV [142]. In pigs, wheat bran supplementation decreases digesta passage rate, increases the abundance of beneficial *Lactobacillaceae*, and elevates SCFAs and peptide YY concentrations. Consequently, dietary fine wheat bran improves satiety and intestinal microbial composition in sows [56]. Studies report that wheat bran increases SCFA concentrations and improves maternal body condition, metabolic status, and inflammatory status by modulating gut microbiota structure in pregnant sows, with increased propionate and butyrate concentrations [143]. Wheat bran significantly enriches *Lactobacillus*, *Bifidobacterium*, and *Faecalibaculum* and alters lipid metabolic pathways, indicating changes in gut microbiota composition and lipid metabolism [90].

Human microbiome studies: Children with wheat allergies show distinct gut microbiome changes, including increased abundances of Firmicutes and Verrucomicrobia and shifts in key genera, such as *Anaerostipes*, *Prevotella*, and *Ruminiclostridium*. In contrast, healthy children exhibit higher abundances of potentially beneficial microbes, including *Megamonas* and *Romboutsia*. Functional analyses further reveal differences in metabolic pathways, particularly those involved in arginine and polyamine biosynthesis [144]. Interindividual variation in microbiota response to wheat-derived fibers is also clinically relevant. Chung et al. [51] show that baseline *Prevotella* abundance determines the bifidogenic response to wheat bran arabinoxylan oligosaccharides in older adults, supporting personalized prebiotic strategies.

Animal and in vitro studies: Additionally, steamed bread made with whole-wheat or aleurone-enriched wheat flour significantly alters hepatic metabolism and modulates gut microbial composition in C57BL/6J mice, suggesting potential as a functional food for preventing high-fat diet-induced metabolic disorders [145]. Similarly, diets supplemented with red wheat and its outer fractions, including dietary fiber and the phenolic-rich aleurone layer, modify gut microbiota composition and alter genera, such as *Lactobacillus*, *Mucispirillum*, *Phascolarctobacterium*, and *Blautia coccoides*, with effects on colon cancer-related biomarkers in rat models [146]. Additionally, anthocyanins from colored wheat sources increase the Bacteroidetes-to-Firmicutes ratio and the abundance of potentially beneficial *Bacteroides* and *Prevotella* [40,147].

Bioprocessed wheat materials exhibit an improved phenolic profile. Colonic microbiota release bound phenolics that escape upper gastrointestinal digestion, potentially contributing to local antioxidant effects and promoting beneficial bacterial populations, including butyrate-producing Firmicutes [27]. Sourdough fermentation modifies the effects of wheat-based bread on the gut microbiome. Using a colon-on-a-plate in vitro model with fecal samples from healthy donors, whole-grain sourdough bread significantly increases SCFA production and alters beneficial microbial taxa compared to refined yeast bread. These findings support the complementary benefits of whole-grain flour and sourdough fermentation [57].

Hydrolyzed and fermented wheat arabinoxylan oligosaccharides enhance SCFA production and *Lactobacillus* abundance more effectively than non-hydrolyzed wheat bran in the simulated human intestinal microbial ecosystem in vitro model [50]. Overall, wheat, particularly as whole grain, biofortified colored varieties, or bioprocessed ingredients (sprouted, fermented, or enzymatically hydrolyzed), represents a versatile dietary substrate for favorable gut microbiota modulation, immune function, and gastrointestinal health. However, gluten and other immunogenic wheat proteins may cause adverse effects in susceptible populations, warranting further consideration.

Evidence Level Summary: Gut microbiome modulation by wheat-derived compounds is supported by Level 2 (observational human studies), Level 3 (animal studies), and Level 4 (in vitro/SHIME models). Distinction between microbiome association and microbiome-mediated causation must be maintained. Table 7 summarizes key studies on wheat-derived modulation of the gut microbiota.

#### 4.7.1. Mechanisms of Gut Microbiota Modulation

Wheat-derived components modulate gut microbiota through multiple interrelated pathways. First, wheat arabinoxylans and β-glucans serve as prebiotic substrates fermented by specific bacterial taxa, particularly *Bifidobacterium* and *Lactobacillus* species, producing SCFAs, including propionate, acetate, and butyrate [56]. Second, microbial esterases release bound phenolic compounds, particularly ferulic acid, during colonic fermentation. These phenolics exert local antioxidant effects and may undergo further metabolism to bioactive metabolites, including dihydroferulic acid and feruloylglycine [27]. Third, wheat-derived alkylresorcinols and benzoxazinoids modulate quorum sensing in pathogenic bacteria, potentially limiting opportunistic pathogen colonization while supporting beneficial commensals [42]. Fourth, wheat peptides directly interact with epithelial TLRs, modulating inflammatory signaling and enhancing tight-junction protein expression [116,148].

#### 4.7.2. Clinical Implications of Gut Microbiome Modulation

Wheat-mediated gut microbiome modulation has significant clinical implications. In individuals with metabolic syndrome, wheat bran consumption increased *Akkermansia muciniphila* abundance and reduced the Firmicutes-to-Bacteroidetes ratio, changes correlating with improved insulin sensitivity and reduced systemic inflammation [140]. In older adults, the prebiotic response to wheat bran arabinoxylan oligosaccharides depends on baseline *Prevotella* abundance, suggesting opportunities for personalized prebiotic interventions [51].

Furthermore, children with wheat allergies exhibit distinct gut microbiome signatures, suggesting the potential use of microbiome profiling as a diagnostic or prognostic tool [144]. Future research should prioritize large-scale randomized controlled studies to establish causal relationships between wheat-induced microbiome changes and clinical outcomes. Although most evidence is preclinical, the health effects of wheat-derived bioactive compounds have been evaluated in several clinical trials (Table 8). Limited clinical trials support the need for large-scale randomized controlled studies to establish definitive health claims for wheat-based functional foods.

### 4.8. Integrated Molecular Mechanisms: Shared Signaling Pathways in Wheat-Mediated Health Effects

Wheat-derived bioactive compounds exert their pleiotropic health effects through modulation of several evolutionarily conserved signaling pathways that are central to cellular stress responses, metabolism, and inflammation. Rather than operating through entirely distinct mechanisms for each health outcome, these compounds converge on a relatively small set of molecular targets that integrate signals across multiple disease contexts.

#### 4.8.1. Nrf2/ARE Antioxidant Pathway

The nuclear factor erythroid 2-related factor 2 (Nrf2) pathway represents the primary cellular defense against oxidative stress. Wheat phenolic compounds, particularly ferulic acid, caffeic acid, and anthocyanins, activate Nrf2 by promoting its dissociation from Keap1, leading to nuclear translocation and transcriptional upregulation of antioxidant response element (ARE)-dependent genes [113]. This results in increased expression of phase II detoxifying enzymes, including heme oxygenase-1 (HO-1), NAD(P)H:quinone oxidoreductase 1 (NQO1), glutathione S-transferases, and superoxide dismutase [60,113]. The Nrf2 pathway mediates antioxidant effects across all disease models examined—reducing oxidative damage in diabetes, protecting against inflammation in colitis models, and preventing carcinogen-induced DNA damage in cancer models [109,113]. Notably, wheat germ oil activates Nrf2/HO-1 signaling in ethanol-induced hepatorenal injury, while wheat bran polyphenols exert protective effects through Nrf2 activation in DSS-induced colitis [109,113].

#### 4.8.2. NF-κB Inflammatory Pathway

Nuclear factor kappa B (NF-κB) is a master regulator of pro-inflammatory gene expression. Wheat-derived compounds, including ferulic acid, alkylresorcinols, and bioactive peptides, inhibit NF-κB activation through multiple mechanisms: preventing IκBα phosphorylation and degradation, reducing p65 nuclear translocation, and suppressing downstream inflammatory gene transcription [109,111,116]. This pathway is central to the anti-inflammatory effects observed across disease contexts: wheat peptides inhibit LPS-induced NF-κB p65 activation in intestinal epithelial cells, reducing TNF-α, IL-6, and IL-1β production [116]; wheat bran polyphenols suppress NF-κB inflammasome pathways in ulcerative colitis [109]; and wheat seedling extracts reduce NF-κB-mediated inflammation in sarcopenia models [110]. The convergence on NF-κB inhibition explains why wheat-derived compounds show efficacy across diverse inflammatory conditions, from metabolic syndrome to autoimmune disorders.

#### 4.8.3. AMPK Metabolic Pathway

AMP-activated protein kinase (AMPK) functions as a cellular energy sensor that coordinates metabolic homeostasis. Wheat-derived compounds, particularly anthocyanins, dietary fibers, and alkylresorcinols, activate AMPK through increased AMP/ATP ratios and upstream kinase signaling [76,110,117]. AMPK activation promotes glucose uptake (GLUT4 translocation), fatty acid oxidation (via PGC-1α), and mitochondrial biogenesis, while inhibiting lipogenesis (via ACC) and gluconeogenesis (via PEPCK and G6Pase) [76,80]. This pathway underlies the antidiabetic and anti-obesity effects of wheat: anthocyanin-rich black wheat activates the adiponectin-AMPK pathway in T2DM rats, improving insulin sensitivity and glucose tolerance [76]; wheat seedling extracts activate AMPK/SIRT3/PGC-1α pathway to ameliorate sarcopenia [110]; and wheat alkylresorcinols regulate muscle fiber type conversion via the miR-34a/SIRT1/AMPK axis [95].

#### 4.8.4. PI3K/AKT Insulin Signaling

The phosphoinositide 3-kinase (PI3K)/AKT pathway is a critical mediator of insulin action and cell survival. Wheat peptides and phenolic compounds enhance PI3K/AKT signaling by increasing insulin receptor (IR) phosphorylation, upregulating insulin receptor substrate (IRS) proteins, and promoting AKT activation at Ser473 [76,81,82,83]. This pathway mediates the antidiabetic effects of wheat: wheat bran anthocyanins restore AKT phosphorylation and glucose uptake in HepG2 cells [81]; black wheat chapatti activates the PI3K/AKT pathway, upregulating GLUT2 and GLUT4 expression in diabetic rats [68]; and wheat germ peptide increases p-AKT/AKT and GLUT2 expression while decreasing SOCS3 and FOXO1 in insulin-resistant HepG2 cells [75]. The PI3K/AKT pathway also contributes to anticancer effects through AKT-mediated regulation of cell survival and apoptosis [129,132].

#### 4.8.5. MAPK Signaling Cascade

Mitogen-activated protein kinase (MAPK) pathways, including ERK, JNK, and p38, mediate cellular responses to stress and inflammatory signals. Wheat-derived compounds modulate MAPK signaling in a context-dependent manner: wheat peptides inhibit LPS-induced MAPK activation in intestinal epithelial cells, reducing inflammatory mediator production [116]; wheat bran polyphenols suppress MAPK/NF-κB inflammasome pathways in colitis models [109]; and ferulic acid activates the ATM/Chk2 and ATR/Chk1 pathways, leading to cell cycle arrest in colon cancer cells [128].

The integration of these shared molecular pathways explains the broad-spectrum health benefits of wheat bioactive compounds. Rather than acting through disease-specific mechanisms, wheat-derived compounds target fundamental cellular stress response and metabolic regulation pathways that are dysregulated across multiple chronic diseases. This convergence has important implications for functional food development—compounds that activate Nrf2 while inhibiting NF-κB and activating AMPK may simultaneously address oxidative stress, inflammation, and metabolic dysfunction, making them attractive candidates for preventing and managing complex chronic diseases.

#### 4.8.6. Summary of Integrated Mechanisms

The integration of these shared molecular pathways explains the broad-spectrum health benefits of wheat bioactive compounds. Rather than acting through disease-specific mechanisms, wheat-derived compounds target fundamental cellular stress response and metabolic regulation pathways that are dysregulated across multiple chronic diseases. This convergence has important implications for functional food development. Compounds that activate Nrf2 while inhibiting NF-κB and activating AMPK may simultaneously address oxidative stress, inflammation, and metabolic dysfunction, making them attractive candidates for preventing and managing complex chronic diseases. Table 9 provides a consolidated summary of shared molecular targets and their associated health outcomes.

### 4.9. Human Evidence and Translational Relevance

This section synthesizes the available human evidence for wheat-derived bioactive compounds, explicitly separating randomized controlled trials (RCTs) from observational studies and reporting effect sizes and uncertainty where available. Whole-grain intake, whole-wheat bread, colored wheat products, wheat bran, wheat germ, fermented wheat germ extract (FWGE), purified compounds, and isolated peptides are treated as distinct interventions. Evidence that whole-grain diets are associated with health outcomes should not be presented as proof that a specific wheat phenolic compound is responsible.

#### 4.9.1. Randomized Controlled Trials

Table 8 summarizes the available RCTs investigating wheat-derived interventions in human populations. The evidence base remains limited, with few large-scale, long-term, placebo-controlled trials available.

#### 4.9.2. Observational Studies

Observational studies have consistently reported associations between whole-grain wheat consumption and reduced risk of chronic diseases. A cohort analysis of 160 participants with diabetes showed that wheat consumption (40 g/day for 30 days) was associated with diabetes management [73]. However, these observational findings do not establish causation, and the effect size of wheat consumption on glycemic outcomes in humans requires confirmation through RCTs.

A meta-analysis of 21 RCTs reports that whole-grain consumption does not consistently affect anthropometric measures, including body weight, BMI, waist circumference, or fat mass [103]. This suggests that the benefits of wheat consumption may depend on the specific wheat fraction involved and may be mediated through mechanisms beyond simple weight loss (e.g., improved insulin sensitivity, reduced inflammation).

#### 4.9.3. Translational Relevance and Knowledge Gaps

Despite substantial preclinical evidence, several critical knowledge gaps limit the translational relevance of wheat-derived bioactive compounds for human health:

(a) Dose–Response Relationships: The doses tested in animal studies often exceed achievable human intake from dietary wheat consumption. The relationship between dietary wheat intake and bioactive compound exposure in humans remains poorly characterized.
(b) Bioavailability and Metabolism: Most wheat phenolics are bound to cell-wall components and require colonic fermentation for bioactivation. The metabolic fate of specific compounds and their contribution to systemic health effects are not well defined.
(c) Individual Variability: Inter-individual variation in gut microbiota composition significantly affects phenolic metabolism and the production of bioactive metabolites, which may contribute to the variable health responses to wheat-based interventions observed in clinical studies [51].
(d) Whole Food vs. Isolated Compounds: Health benefits attributed to whole-grain wheat consumption may not be replicable with isolated phenolic compounds or purified fractions. The complex phytochemical matrix in whole wheat likely produces synergistic effects that differ from isolated compounds.
(e) Long-Term Safety: The long-term safety and efficacy of high-dose wheat bioactive compound supplementation in humans remains unestablished. Most studies have been short-term (weeks to months), and the potential for adverse effects from prolonged high-dose consumption of specific wheat-derived compounds has not been adequately assessed.

#### 4.9.4. Summary of Human Evidence

The human evidence for wheat-derived bioactive compounds remains limited, with few large-scale, long-term RCTs available. While observational studies suggest associations between whole-grain wheat consumption and reduced chronic disease risk, these findings require confirmation through well-designed RCTs. The translation of preclinical findings to clinical practice requires:

(a) Larger, longer-term RCTs with standardized interventions and outcomes;
(b) Mechanistic studies in humans to confirm pathway engagement;
(c) Identification of biomarkers of response to wheat-based interventions;
(d) Assessment of inter-individual variability and predictors of response;
(e) Evaluation of safety and tolerability in diverse populations.

Clinicians and nutrition researchers should be cautious in extrapolating preclinical findings to clinical recommendations. Whole-grain wheat consumption as part of a balanced diet is consistent with dietary guidelines, but specific health claims for isolated wheat-derived compounds require further validation in human studies.

### 4.10. Safety Considerations: Gluten-Related Disorders

#### 4.10.1. Celiac Disease

While wheat offers numerous health benefits, gluten and other wheat proteins can trigger adverse reactions in susceptible individuals. Recognizing both the beneficial and potentially adverse effects of wheat consumption is essential for developing comprehensive nutritional recommendations and wheat-based functional foods.

Celiac disease (CeD), an autoimmune disorder, affects approximately 1% of the global population. Gluten ingestion causes the disease in genetically predisposed individuals carrying the HLA-DQ2 or HLA-DQ8 haplotypes [54]. Tissue transglutaminase deamidates gluten-derived peptides, particularly those from α-, γ-, and ω-gliadins, subsequently presented to T cells, triggering an inflammatory response that damages the small intestinal mucosa. Clinical manifestations include diarrhea, malabsorption, weight loss, anemia, dermatitis herpetiformis, and neurological symptoms. Currently, lifelong strict adherence to a gluten-free diet remains the only established treatment for CeD (Table S4).

Evidence level: Level 1 (clinical guidelines) and Level 2 (observational human studies).

#### 4.10.2. Non-Celiac Gluten Sensitivity

Non-celiac gluten sensitivity (NCGS) is a distinct clinical condition characterized by intestinal and extraintestinal symptoms following gluten ingestion, without evidence of CeD or wheat allergy [150]. The estimated prevalence of NCGS ranges from 0.5% to 13% of the general population; however, accurate epidemiological data remain limited, as no specific biomarker is available. Symptoms include abdominal pain, bloating, fatigue, headache, and brain fog and typically resolve within hours to days after gluten withdrawal. The pathophysiology of NCGS remains unclear but may involve innate immune activation, increased intestinal permeability, and direct toxic effects of gluten peptides on enterocytes (Table S4). Evidence level: Level 2 (observational human studies).

#### 4.10.3. Wheat Allergy

Wheat allergy, an immunoglobulin E (IgE)-mediated allergic reaction to wheat proteins (e.g., gluten, albumin, and globulin fractions), affects approximately 0.2–1% of children; however, many affected children outgrow the condition by school age [151]. Clinical presentations include mild oral allergy syndrome, urticaria, and severe anaphylaxis. Wheat-dependent exercise-induced anaphylaxis is a specific form of wheat allergy characterized by symptoms occurring only when wheat intake is followed by physical exertion. Management requires strict avoidance of wheat-containing foods and access to epinephrine for severe reactions (Table S4). Evidence level: Level 2 (observational human studies).

#### 4.10.4. Gluten-Related Disorders Overview

Gluten-related disorders (GRDs) encompass CeD, NCGS, and wheat allergy, and rarer conditions, such as gluten ataxia and dermatitis herpetiformis. Global GRD burden has increased in recent decades, potentially reflecting greater diagnostic awareness, changes in wheat processing and consumption patterns, and environmental factors [54].

#### 4.10.5. Breeding Strategies to Reduce Immunogenicity

Recent advances in plant biotechnology offer promising strategies for developing wheat varieties with reduced immunogenic potential while retaining beneficial bioactive compounds. A total 97.7% reduction in the gluten content was observed in bread wheat using multiplex CRISPR/Cas9 editing of the α-, γ-, and ω-gliadin genes. The edited wheat lines show significantly reduced T-cell reactivity in CeD patient-derived T-cell lines [152]. Similarly, Marin-Sanz and Barro [153] developed ultra-low-gliadin wheat using an integrated RNA interference (RNAi), CRISPR, and doubled-haploid technology, eliminating immunodominant gliadin epitopes while maintaining acceptable baking quality.

However, several critical limitations must be acknowledged:

(a) Regulatory Hurdles: Genetically modified wheat varieties face significant regulatory barriers, particularly in jurisdictions with strict GMO regulations.
(b) Baking Quality: The impact of gluten reduction on bread-making quality (dough rheology, loaf volume, crumb structure) requires careful optimization.
(c) Consumer Acceptance: Consumer acceptance of genetically modified food products remains a significant barrier.
(d) Long-Term Safety: The long-term health effects of consuming reduced-gluten or gluten-free wheat varieties require further investigation.
(e) Contamination Control: Field stability and contamination control present practical challenges for commercial production.

#### 4.10.6. Clinical and Nutritional Implications

Individuals with confirmed CeD, wheat allergy, or severe NCGS should strictly avoid gluten-containing wheat products. However, whole-grain wheat consumption in the general population without these conditions is associated with a lower risk of chronic disease. Food scientists and nutrition researchers must develop wheat-based products safe for gluten-sensitive individuals while retaining health-promoting bioactive compounds.

Potential strategies include:

(a) Low-gluten genetically modified wheat (requires regulatory approval and safety testing);
(b) Enzymatic gluten degradation during food processing (using prolyl endopeptidases);
(c) Wheat-derived extracts enriched in phenolic compounds and depleted of immunogenic proteins;
(d) Germinated or fermented wheat products with reduced gluten content (though not gluten-free).

Any low-gluten or edited wheat discussed for sensitive populations requires rigorous immunogenicity testing, regulatory assessment, field stability, contamination control, and clinical evaluation before being considered safe for individuals with celiac disease.

## 5. Artificial Intelligence-Guided Chemical Profiling of Wheat

Recent studies show that AI, particularly machine and deep learning, is a valuable tool in food science, phytochemical screening, and biological data analysis [154]. In wheat research, AI has been increasingly used to predict bioactive compounds and nutraceuticals and to assess the nutritional and quality characteristics of wheat flour. Critically, AI serves as a translational bridge between the phytochemical characterization described in Section 2, Section 3 and Section 4 and the practical development of wheat-based functional foods. By integrating high-throughput spectroscopic data with ML algorithms, AI enables rapid, non-destructive prediction of bioactive compound profiles that would otherwise require time-consuming laboratory analysis.

Conventional chemical profiling of wheat relies on laboratory-based techniques including high-performance liquid chromatography (HPLC), gas chromatography–mass spectrometry (GC-MS), and liquid chromatography–mass spectrometry (LC-MS). While these methods provide accurate results, they are often time-consuming, destructive, and costly for large-scale wheat profiling. Integrating AI and ML with spectroscopic and imaging techniques offers opportunities for high-throughput, non-destructive chemical profiling of wheat bioactive compounds, nutraceutical constituents, and nutritional characteristics.

The AI section distinguishes three distinct applications:

(a) AI for prediction of measurable composition or quality traits (Section 5.1);
(b) AI for classification or screening of wheat materials (Section 5.2);
(c) AI for discovery of bioactive molecules or targets (Section 5.3).

Table 10 summarizes AI-based techniques used for chemical profiling in wheat across various studies discussed below.

### 5.1. Artificial Intelligence-Guided Detection and Profiling of Bioactive Compounds

Researchers applied quantum-chemical descriptors to wheat phenolic compound analysis, integrating quantitative structure–activity relationship (QSAR) modeling with a genetic algorithm for molecular docking and feature selection to characterize phenolic acid binding to β-cyclodextrin in wheat bran. The model achieved R^2^ values of 0.969 and 0.984 during training and testing, respectively. Aromaticity, electronegativity, and polar surface area influenced molecular binding [155]. An extended model was developed for classifying wheat phenolic compounds across different thermal treatments by combining visible–near-infrared–short-wave infrared (Vis-NIR-SWIR) spectroscopy with random forest (RF), K-nearest neighbors (KNN), and decision tree (DT) classifiers. The RF model achieved 0.98–0.99 accuracy using the proposed approach. Explainable AI (XAI) identified key spectral wavelengths supporting interpretability and phenolic compound classification for food quality applications [156]. Weighted gene co-expression network analysis (WGCNA) integrated with transcriptomic data identified the flavonoid biosynthesis pathway as a core molecular mechanism underlying heat tolerance in wheat [157].

Critical Assessment: The accuracy values reported (0.98–0.99) are promising but require cautious interpretation. These models were developed on limited sample sets and may not generalize to diverse wheat varieties, growing conditions, or processing methods. External validation on independent datasets from different cultivars, growing locations, harvest years, instruments, and laboratories is needed before these models can be considered robust for commercial applications.

### 5.2. Artificial Intelligence-Guided Nutritional Composition Analysis

Traditional methods for assessing wheat quality parameters, including protein, gluten, starch, moisture, and mineral content, are limited by low throughput. AI-driven hyperspectral imaging and NIR spectroscopy offer a shift toward high-throughput, nondestructive nutritional profiling.

A gluten detection approach for managing celiac disease used an Internet of Things (IoT) platform combining portable NIR spectroscopy with ML and DL algorithms. The system was evaluated using 12,000 corn, rye, and oat flour samples and achieved an F2 score of 96.06%. The end-to-end model required only 1 min, supporting high-potential edge-computing NIR devices for food safety screening [158]. Using 402 global wheat varieties, a model simultaneously predicted >220 nutritional components including lipids, proteins, amino acids, vitamins, polyphenols, and minerals. Vis-NIR hyperspectral imaging combined with stepwise linear regression enabled robust quantification. A DL pixel-to-pixel generative adversarial network (GAN) model mapped the spatial distribution of nutrients within wheat, achieving high external fidelity = 0.99 [156,159].

Hyperspectral imaging and Fourier-transform infrared (FTIR) spectroscopy data were integrated to train RF, DT, and KNN classifiers for nondestructive gluten-strength assessment. The models achieved prediction accuracies >0.98 for hyperspectral features and 0.99 for Mixolab features, with validation using seven commercial flour blends [160]. A portable NIR spectroscopy method was developed for simultaneous quantification of gluten index (GI), dry gluten content, and wet gluten content in wheat flour. Among evaluated ML algorithms including support vector regression (SVR), RF, artificial neural network (ANN), gradient boosting machine, and partial least squares (PLS), SVR achieved the best performance with R^2^ *p* = 0.93–0.94 and relative predictive deviation = 3.13–3.50 [161]. A multi-output ANN model was developed for predicting physiological, nutritional, and biochemical parameters in durum wheat exposed to NaCl-induced salinity stress and biostimulant treatment [162].

LGAKNet, a lightweight real-time network for monitoring wheat flour, integrates Ghost bottlenecks, an external attention module, and a Kolmogorov–Arnold network (KAN) to process NIR spectra. The model was evaluated using 519 samples, achieving high accuracy for protein and moisture prediction. Class activation mapping (CAM) supported model interpretability by identifying decision-relevant C-H/N-H and O-H water absorption bands [163]. RF regression using Vis-NIR hyperspectral data achieved an R^2^ value of 0.8579 for wet gluten prediction. Absorption features at 450 nm (color) and 930 nm (C-H protein overtone) were identified as critical spectral regions for gluten prediction [164].

Critical Assessment: While these models show promising predictive performance, several methodological limitations must be acknowledged:

(a) Sample-to-feature ratio is often inadequate for robust model development;
(b) Train/validation/test partitioning and cross-validation strategies are not always clearly reported;
(c) Potential leakage from splitting spectra from the same batch across training and test sets;
(d) Limited external validation on independent datasets from different cultivars, locations, and instruments;
(e) Lack of confidence intervals and calibration reporting.

The distinction between internal and external validation is critical. Where multiple spectra or image pixels from the same grain, flour batch, field, or cultivar were split across training and test sets, this can lead to optimistic performance estimates. Test sets should be held out before model development, and external validation should represent different cultivars, growing locations, harvest years, instruments, and laboratories. Reporting consistent with TRIPOD+AI principles would substantially improve transparency and reproducibility.

### 5.3. Artificial Intelligence-Guided Discovery of Nutraceutical Compounds

Beyond conventional wheat-component quantification, AI can facilitate the de novo identification of novel bioactive compounds and nutraceuticals. Umami peptides from fermented wheat gluten hydrolysates were identified using ML algorithms including IUmami-SCM, Taste Peptides DM, Umami-YYDS, and UMPred-FRL, combined with molecular docking against the T1R1/R1R3 receptor. Six novel peptides (LSFE, QQLPQFEE, YTCE, EELR, YTTD, and EEDQ) were identified with stable, high-affinity binding ranging from −6.3 to −8.1 kcal/mol. Subsequent sensory tests confirmed stronger umami activity than monosodium glutamate (MSG), with EEDQ showing the greatest taste activity [165]. A DL and molecular dynamics model was developed to screen 2798 wheat gliadin-derived peptides for interactions with the calcium-sensing receptor. This AI-based approach identified the lead peptide RLSYQFPFYP with strong binding affinity (Kd = 74.4 nM). Experimental results showed that the peptide enhanced antioxidant activity and reduced inflammatory markers including TNF-α, IL-8, and IL-6 [148].

Critical Assessment: Docking and virtual screening are hypothesis-generating tools. They do not, by themselves, establish receptor engagement in a biological system, pharmacokinetics, oral stability, intestinal absorption, safety, or clinical benefit. For the peptide studies reviewed, the following limitations must be acknowledged:

(a) Peptide purity and sequence confirmation are not always specified;
(b) Digestion stability in gastrointestinal conditions requires further investigation;
(c) Dose–response relationships and cytotoxicity need evaluation;
(d) Receptor selectivity has not been established;
(e) Independent replication in other laboratories is needed.

The term “nutraceutical discovery” should be reserved for candidates that have progressed beyond computational prioritization. AI-based identification represents an early-stage screening tool that generates hypotheses for experimental validation. Table 10 summarizes AI-based techniques used for chemical profiling in wheat across various studies discussed in Section 5.1, Section 5.2 and Section 5.3, with explicit reporting of sample sizes, validation approaches, and key limitations.

### 5.4. AI-Multi-Omics Integration for Personalized Wheat-Based Nutrition

The convergence of AI with multi-omics technologies is enabling a paradigm shift toward personalized nutrition using wheat-derived bioactives. This integrated approach addresses a fundamental limitation of conventional nutritional recommendations, the significant inter-individual variability in response to dietary interventions, by identifying biomarkers that predict individual responses to wheat-based functional foods.

Genomics and Transcriptomics: AI-integrated genomic and transcriptomic analyses enable the identification of wheat varieties with optimal bioactive profiles for specific health applications. Shen et al. [157] integrated WGCNA with transcriptomic data to identify the flavonoid biosynthesis pathway as a core molecular mechanism underlying heat tolerance in wheat, revealing genotype-specific flavonoid profiles that could be targeted for functional food development. Transcriptomic analysis of colored wheat varieties has identified key regulatory genes (TaCHI, TaDFR, TaF3H, TaUGT, TaANS, TaMT) whose expression correlates with anthocyanin content, enabling AI-assisted prediction of bioactive compound accumulation based on gene expression signatures [1]. For personalized applications, an individual’s genetic variants in metabolic pathways (e.g., polymorphisms in FTO, TCF7L2, or PPARG genes) could inform the selection of wheat varieties or bioactive extracts tailored to their metabolic phenotype.

Metabolomics and Chemical Profiling: AI-powered metabolomics provides high-resolution characterization of wheat bioactive compound profiles across varieties, growing conditions, and processing methods. The combination of Vis-NIR hyperspectral imaging with DL (pix2pix GAN) enabled simultaneous quantification and spatial visualization of >220 nutritional components across 402 global wheat accessions [159]. This capability, when integrated with individual metabolomic profiles, could enable personalized matching of wheat varieties to specific nutritional needs—for example, selecting anthocyanin-rich black wheat for individuals with insulin resistance or high-fiber bran fractions for those with dyslipidemia.

Microbiome Profiling: The gut microbiome plays a critical role in mediating the health effects of wheat bioactive compounds, particularly for bound phenolics that require colonic fermentation for bioactivation [27]. Chung et al. [51] demonstrated that baseline Prevotella abundance determines the bifidogenic response to wheat bran arabinoxylan oligosaccharides in older adults, establishing a clear biomarker for personalized prebiotic strategies. AI-driven microbiome analysis can identify individuals who would benefit most from specific wheat fractions based on their microbial capacity to metabolize bound phenolics, produce SCFAs, or generate bioactive metabolites [57]. This approach enables the development of “microbiome-matched” functional foods, where wheat-based products are tailored to individual gut microbial communities.

Integrated framework for personalized wheat-based functional foods: The integration of these omics layers with AI creates a comprehensive framework for personalized wheat-based nutrition. A conceptual workflow involves: (1) individual genomic, metabolomic, and microbiome profiling to establish baseline characteristics; (2) AI-integrated multi-omics analysis to predict individual responses to specific wheat bioactive compounds, drawing on large datasets linking omics signatures to health outcomes; (3) AI-assisted selection of optimal wheat varieties, fractions, or bioactive extracts matched to individual profiles; (4) real-time monitoring of response through non-destructive spectroscopy integrated with AI; and (5) iterative refinement of recommendations through ML algorithms that learn from individual responses over time.

This personalized approach addresses the substantial inter-individual variation observed in clinical trials of wheat-based interventions [51,103]. While current evidence is primarily preclinical or based on small cohort studies, the rapid advancement of AI-integrated multi-omics technologies provides a clear pathway toward precision nutrition using wheat-based functional foods, with the potential to maximize health benefits while minimizing adverse effects in susceptible populations.

### 5.5. Limitations and Future Directions for AI in Wheat Research

Several methodological limitations must be acknowledged when interpreting AI-based studies in wheat research:

(a) Sample Size and Representativeness: Many studies use limited sample sets that do not capture the full diversity of wheat varieties, growing conditions, and processing methods. Models developed on narrow datasets may not generalize to broader applications.
(b) Data Leakage: Splitting multiple spectra or image pixels from the same grain, flour batch, field, or cultivar across training and test sets can lead to optimistic performance estimates. Test sets should be held out before model development.
(c) External Validation: The distinction between internal and external validation is critical. External validation on datasets representing different cultivars, growing locations, harvest years, instruments, and laboratories is needed before models can be considered robust for commercial applications.
(d) Model Interpretability: While XAI approaches (e.g., SHAP, LIME, CAM) improve interpretability, the biological relevance of identified spectral features requires independent validation.
(e) Reporting Standards: Many studies do not consistently report sample numbers, sample-to-feature ratio, preprocessing steps, batch structure, train/validation/test partitioning, cross-validation strategy, hyperparameter tuning, feature selection, leakage prevention, confidence intervals, baseline models, calibration, or external validation. Reporting consistent with TRIPOD+AI principles would substantially improve transparency and reproducibility.
(f) Translation to Practice: High accuracy in predicting flour protein or gluten index does not demonstrate discovery of a nutraceutical compound, and molecular docking affinity does not establish biological efficacy. AI-based identification represents an early-stage screening tool that generates hypotheses for experimental validation.

#### Future Directions

Future AI applications in wheat research should prioritize:

(a) Large-scale, multi-center datasets: Development of publicly available, standardized datasets encompassing diverse wheat varieties, growing conditions, processing methods, and geographical locations to enable robust model development and validation.
(b) Prospective validation studies: AI models should be validated in prospective studies with clearly defined outcomes, rather than retrospective analyses of existing datasets.
(c) Integration of multi-omics data: AI models that integrate genomic, transcriptomic, metabolomic, and microbiome data will enable more comprehensive understanding of wheat bioactive compounds and their health effects.
(d) Explainable and interpretable AI: Development of models that provide biological insights, not just predictions, will accelerate translation to clinical and industrial applications.
(e) Regulatory and safety considerations: AI-based predictions of bioactive compounds and their health effects should be subject to rigorous validation before translation to clinical recommendations or commercial products.
(f) Quantum computing: While quantum computing has been proposed as a theoretical future direction for computational food science, current applications in wheat chemical profiling remain hypothetical and require validation. At present, no established quantum computing or quantum machine learning model exists for wheat bioactive compound discovery or quality prediction. This represents an emerging area that may offer future opportunities, but substantial foundational research is needed before practical applications can be realized.

## 6. Impact of Food Processing on Wheat Bioactive Compounds and Functional Properties

Food processing significantly influences the content, bioavailability, and biological activity of wheat bioactive compounds, while also affecting gluten structure and immunogenicity. Understanding these effects is essential for optimizing processing conditions to maximize nutraceutical potential while maintaining product quality and safety.

### 6.1. Thermal Processing

Baking, extrusion, steaming, and other thermal treatments exert complex effects on wheat bioactive compounds. Moderate thermal processing can enhance phenolic compound extractability by disrupting cell wall structures and releasing bound phenolics from arabinoxylan ester linkages [24]. However, excessive heat causes thermal degradation of heat-labile compounds, particularly anthocyanins (a subclass of flavonoids), which show significant losses (up to 60–80%) during high-temperature baking [1]. Ferulic acid, the predominant wheat phenolic, shows greater thermal stability, with retention rates of 65–85% during bread baking [26].

Thermal processing also impacts gluten structure and digestibility. High-temperature baking (≥200 °C) induces Maillard reactions between reducing sugars and gluten proteins, generating advanced glycation end-products that may alter immunogenicity and nutrient bioavailability [54]. Conversely, controlled thermal treatment can reduce gluten immunoreactivity through structural modification, though complete elimination of immunodominant epitopes requires more intensive processing.

Limitations: The primary limitation of thermal processing is the trade-off between enhancing bioaccessibility and degrading heat-labile bioactive compounds. Optimization requires balancing temperature, time, and moisture content, which varies substantially across wheat varieties and processing objectives. Additionally, thermal processing may generate undesirable by-products (e.g., acrylamide, furans) that require careful monitoring.

### 6.2. Germination and Sprouting

Germination (sprouting) activates endogenous enzymes (β-glucosidases, esterases, xylanases) that hydrolyze ester-linked bound phenolics, releasing free phenolic acids and increasing soluble phenolic content [27,61]. Ultrasound-assisted germination increases gamma-aminobutyric acid (GABA) content to 18 mg/100 g and flavonoid content to 0.19 mg quercetin equivalent (QE)/g, with corresponding increases in antioxidant capacity [61]. Germination temperature and duration positively influence the accumulation of soluble phenolic acids, flavone C-glycosides, and lignans [27]. Sprouted wheat flour improves the phenolic profile and bread-making performance, with enhanced free radical scavenging activity [34].

However, germination also activates proteolytic enzymes that partially degrade gluten proteins, potentially affecting baking quality and dough structure. Extended germination (>72 h) may lead to excessive starch degradation, reducing product quality. Germination conditions must be carefully controlled to maximize bioactive enhancement while maintaining functional properties.

Limitations: Germination requires precise control of temperature, humidity, and duration, with optimal conditions varying across wheat varieties. The process is time-consuming (typically 24–96 h) and may not be economically feasible for large-scale commercial production. Additionally, germinated wheat products may have altered sensory characteristics (e.g., increased bitterness) that limit consumer acceptance.

### 6.3. Fermentation and Bioprocessing

Sourdough fermentation and microbial bioprocessing enhance the release of bound phenolics and generate novel bioactive compounds. Fermentation with *Saccharomyces cerevisiae* and *Bacillus subtilis* modifies wheat bran polysaccharide molecular weight, enhances solubility, and releases bound phenolics, resulting in increased DPPH, hydroxyl, and superoxide radical scavenging activity in chemical assays [43]. Fermented wheat germ extract (FWGE) shows superior anti-inflammatory and anti-obesity effects compared to unfermented germ in animal models, attributed to increased bioavailability of bioactive quinones (2-methoxy-1,4-benzoquinone, 2,6-dimethoxy-1,4-benzoquinone) [93,120]. Sourdough fermentation also partially degrades gluten proteins through proteolytic activity, potentially reducing immunogenicity in susceptible individuals [57].

Fermentation by specific Lactobacillus and Bifidobacterium strains can enhance phenolic bioavailability and generate bioactive metabolites, including short-chain fatty acids (SCFAs) and enterolignans [27,56]. In the colonic environment, resident microbiota ferment indigestible wheat fibers and release bound phenolics, contributing to local antioxidant effects and promoting beneficial bacterial populations [27].

Limitations: Fermentation is a complex process requiring careful selection of microbial strains, optimization of fermentation parameters, and prevention of contamination. The process can produce undesirable flavors or off-notes depending on strain and conditions. Scale-up from laboratory to industrial production presents challenges in maintaining consistent product quality. Additionally, although fermentation partially degrades gluten, it cannot reliably produce gluten-free products safe for celiac patients, necessitating supplementary enzymatic or physical removal methods.

### 6.4. Enzymatic Processing

Enzymatic hydrolysis with specific enzymes (ficin, xylanase, β-glucanase, esterases) offers precise control over bioactive compound release. Ficin hydrolysis of wheat gluten improves dough properties and texture while releasing bioactive peptides with notable antioxidant activity in chemical assays [67]. Xylanase treatment releases feruloyl arabinoxylan oligosaccharides with enhanced antioxidant and anti-inflammatory activity in lipopolysaccharide (LPS)-stimulated macrophages [49,50]. Enzyme selection and hydrolysis conditions significantly affect the phenolic profile: xylanase combined with esterase releases higher levels of free ferulic acid than either enzyme alone [49].

Limitations: Enzymatic processing can generate bitter peptides requiring additional processing steps (e.g., activated carbon treatment, enzymatic debittering). The high cost of commercial enzymes may limit economic feasibility for large-scale production. Enzymes may not completely hydrolyze all bound phenolic compounds, particularly those embedded in complex cell wall structures. Additionally, enzymatic treatment may alter product texture and sensory properties, requiring optimization for specific applications.

### 6.5. High-Pressure and Emerging Technologies

Steam explosion treatment of black wheat bran at 1.0 MPa for 90 s significantly improves soluble fiber, phenolics, flavonoids, and anthocyanin content, enhancing antioxidant activity in chemical assays (DPPH 37.5%, ABTS 31.83%, FRAP 45.82%) [65]. High-pressure processing (HPP) modifies gluten structure and polyphenol interactions without thermal degradation, potentially improving functional properties while reducing immunogenicity [139]. Extrusion cooking, a widely used industrial process, can enhance phenolic bioaccessibility through cell wall disruption but may also degrade heat-labile anthocyanins depending on processing conditions.

Limitations: High-pressure processing equipment is expensive, and the technology may not be economically viable for large-scale production. Steam explosion requires specialized equipment and careful control of pressure, temperature, and exposure time; excessive treatment can degrade bioactive compounds. The scalability of emerging technologies for industrial application remains to be demonstrated, and the effects on product sensory properties require thorough evaluation.

### 6.6. Gluten Modification Technologies

Recent advances in biotechnology offer promising strategies for reducing gluten immunogenicity. CRISPR/Cas9-mediated multiplex editing of α-, γ-, and ω-gliadin genes achieved a 97.7% reduction in gluten content in bread wheat, with significantly reduced T-cell reactivity in celiac disease patient-derived T-cell lines [152]. Integrated RNAi, CRISPR, and doubled-haploid technology produced ultra-low-gliadin wheat with eliminated immunodominant epitopes while maintaining acceptable baking quality [153]. Enzymatic gluten degradation during processing (using prolyl endopeptidases) and extraction of wheat-derived extracts enriched in phenolic compounds while depleted of immunogenic proteins represent additional strategies for developing products safe for gluten-sensitive individuals.

Limitations: Regulatory hurdles significantly delay the commercialization of genetically modified wheat varieties, particularly in jurisdictions with strict GMO regulations. The impact of gluten reduction on bread-making quality (dough rheology, loaf volume, crumb structure) requires careful optimization. Consumer acceptance of genetically modified food products remains a significant barrier. Additionally, the long-term health effects of consuming reduced-gluten or gluten-free wheat varieties need further investigation.

### 6.7. Summary of Processing Effects

Table S5 summarizes the effects of different processing methods on wheat bioactive compounds and gluten, including current limitations that need to be addressed for commercial application. The optimal processing strategy depends on the target compounds, desired bioactivity, product application, and economic feasibility. A combination of processing methods (e.g., germination followed by fermentation or enzymatic hydrolysis) may achieve synergistic enhancement of bioactive compound release and functional properties.

## 7. Conclusions

Wheat is both a globally important staple crop and a valuable source of diverse bioactive compounds with significant nutraceutical potential. Wheat-derived phenolic acids, flavonoids, lignans, alkylresorcinols, phytosterols, dietary fibers, and bioactive peptides exhibit diverse biological activities in preclinical models, including antioxidant, antidiabetic, anti-obesity, anti-inflammatory, anticancer, antimicrobial, and gut microbiota-modulating effects. These health benefits are mediated through convergence on several evolutionarily conserved signaling pathways: Nrf2/ARE for antioxidant defense, NF-κB for inflammation control, AMPK for metabolic regulation, PI3K/AKT for insulin signaling, and MAPK for stress responses, which explains the pleiotropic effects of wheat compounds across multiple disease contexts. However, wheat also contains gluten, which can trigger celiac disease, non-celiac gluten sensitivity, and wheat allergy in susceptible individuals. Therefore, the health effects of wheat consumption are context-dependent and vary across populations, necessitating careful consideration of individual risk factors and the development of wheat products that maximize benefits while minimizing adverse effects.

Wheat bioactive compounds show promise for preventing and managing chronic metabolic and inflammatory diseases in preclinical models. Recent advances in artificial intelligence (AI) have significantly improved phytochemical profiling, bioactivity assessment, and quality prediction, accelerating nutraceutical discovery and functional food innovation. Processing strategies, including germination, fermentation, enzymatic hydrolysis, and emerging technologies like high-pressure processing and CRISPR-mediated genome editing, offer opportunities to enhance bioactive compound release and bioactivity while potentially reducing gluten immunogenicity, though each approach has limitations that must be addressed for commercial application. However, the clinical translation of these findings requires rigorous human studies, and safety considerations for gluten-sensitive populations must be addressed.

### 7.1. Unsolved Scientific Questions

Despite substantial progress, several critical questions remain unresolved:

First, the precise molecular mechanisms underlying synergistic versus antagonistic interactions among wheat bioactive compounds are poorly understood. Most studies examine single compounds or crude extracts, but the complex phytochemical matrix in whole wheat likely produces effects that differ from isolated compounds. Understanding these interactions is essential for rational functional food development.

Second, inter-individual variability in response to wheat-based interventions needs systematic investigation. The role of gut microbiome composition, host genetics (particularly polymorphisms in genes affecting metabolism, inflammation, and immune response), and environmental factors in determining individual responses remains poorly characterized. This knowledge gap limits the development of personalized wheat-based nutrition strategies.

Third, the bioavailability of bound phenolic compounds after colonic fermentation and their tissue-specific distribution and bioactivity require further elucidation. While it is established that colonic microbiota release bound phenolics, the metabolic fate of specific compounds and their contribution to systemic health effects are not well defined.

Fourth, the long-term safety and efficacy of high-dose wheat bioactive compound supplementation in humans remains unestablished. Most studies have been short-term (weeks to months), and the potential for adverse effects from prolonged high-dose consumption of specific wheat-derived compounds has not been adequately assessed.

Fifth, the optimal processing conditions that maximize bioactive compound retention while minimizing anti-nutritional factors and allergenicity need systematic optimization. The trade-offs between enhancing bioaccessibility and degrading heat-labile compounds require resolution through integrated processing strategies.

Sixth, whether genetically modified low-gluten wheat varieties can retain full health-promoting bioactive profiles while reducing immunogenicity remains to be demonstrated in clinical trials. The impact of genetic modification on the complex phytochemical matrix of wheat has not been fully characterized.

### 7.2. Future Research Directions

Addressing these unresolved questions will require coordinated efforts across multiple research domains:

(a) Large-Scale Clinical Trials: Large-scale RCTs are critically needed to establish definitive health claims for wheat-based functional foods. Such trials should incorporate biomarker assessments, microbiome analysis, and genetic profiling to identify response predictors. The distinction between whole-grain dietary patterns, specific wheat fractions, and isolated bioactive compounds must be maintained.
(b) AI-Integrated Multi-Omics: AI-integrated multi-omics approaches should be applied to identify individual response biomarkers and develop personalized wheat-based nutrition strategies. The integration of genomics, transcriptomics, metabolomics, and microbiome profiling with machine learning algorithms offers the potential to predict individual responses and optimize wheat-based interventions.
(c) Advanced Computational Approaches: Quantum computing and advanced AI models may accelerate de novo discovery of novel bioactive peptides, prediction of compound interactions, and identification of molecular targets, warranting continued investment in computational approaches. However, current applications remain hypothetical and require foundational research.
(d) Sustainable Processing Technologies: Sustainable processing technologies that minimize bioactive compound degradation while enhancing bioaccessibility should be prioritized. This includes developing bioprocessing strategies (germination, fermentation, enzymatic hydrolysis) that are scalable, cost-effective, and environmentally sustainable.
(e) Adjunct Therapy Evaluation: The potential of wheat-derived compounds as adjunct therapies for chronic diseases (diabetes, obesity, cancer, inflammatory disorders) warrants clinical investigation. Combination therapies integrating wheat bioactive compounds with conventional pharmaceuticals should be explored, with careful assessment of interactions and synergistic effects.
(f) Microbiome–Health Axis: The interplay between wheat polyphenols and the gut microbiome in mediating systemic health effects should be explored through longitudinal human intervention studies with comprehensive microbiome, metabolome, and immune function analyses.
(g) Safety and Regulatory Science: Comprehensive safety assessment of concentrated wheat extracts, fermented products, high-dose peptides, and genetically modified or gene-edited wheat is needed before clinical translation. Regulatory frameworks for wheat-based functional foods and nutraceuticals require development.
(h) Evidence Synthesis and Standardization: Future reviews should adopt systematic methodology with explicit evidence hierarchies, transparent reporting of the literature search and selection, and calibrated language distinguishing hypothesis-generating evidence from validated human outcomes. Reporting of AI-based prediction studies should follow TRIPOD+AI principles.

### 7.3. Concluding Remarks

In conclusion, wheat shows strong potential for developing next-generation functional foods and nutraceuticals. The convergence of wheat science, processing technology, AI, and personalized nutrition represents an exciting frontier with the capacity to transform wheat from a staple grain into a precision therapeutic platform for chronic disease prevention and management.

However, realizing this potential requires addressing the unresolved scientific questions and pursuing the outlined research directions. Critical gaps remain in understanding synergistic compound interactions, inter-individual variability, bioavailability, long-term safety, optimal processing conditions, and clinical efficacy of reduced-gluten wheat varieties. The evidence for health benefits remains primarily preclinical, with limited human data available. Clinicians and nutrition researchers should be cautious in extrapolating preclinical findings to clinical recommendations.

Integrating innovative computational technologies with nutritional and phytochemical research provides a forward-looking framework for enhancing human health and advancing precision healthcare through wheat-based dietary interventions. Whole-grain wheat consumption as part of a balanced diet is consistent with dietary guidelines, but specific health claims for isolated wheat-derived compounds require further validation in human studies.

## Figures and Tables

**Table 1:** Bioactive compounds and distribution in wheat grain fractions.

Grain Fraction	Proportion (% of Kernel)	Main Components	Key Bioactive Compounds	Concentration Range	Primary Health Implications	Refs.
Starchy endosperm	80–85	Starch, storage proteins	Low phenolics, vitamin E, and carotenoids	Phenolic acids: 50–150 μg/g; vitamin E: 0.1–0.3 mg/100 g	Primary energy source; limited bioactive contribution	[47,52,53]
Aleurone layer	6–9	Proteins, enzymes, fiber	Arabinoxylans, β-glucans, ferulic acid, *p*-coumaric acid, sinapic acid, apigenin, luteolin, lignans, alkylresorcinols, benzoxazinoids, tocopherols, tocotrienols, B vitamins, minerals, phytic acid, phytosterols	Phenolic acids: 300–800 μg/g; dietary fiber: 30–45%; B vitamins: 3–10 mg/100 g	High fiber and phenolic content; fermentation in the colon produces SCFAs; contributes to metabolic health	[35,47,52,53]
Germ (embryo)	3	Lipids, enzymes	Tocopherols, tocotrienols, essential fatty acids, carotenoids, sterols, phenolic compounds, B vitamins	Phenolic acids: 150–350 μg/g; vitamin E: 10–30 mg/100 g; protein: 25–30%; lipids: 8–12%	Rich source of bioavailable soluble phenolics, essential fatty acids, and vitamin E; contributes to antioxidant defense and cardiovascular health	[47,48,52,53]
Testa (seed coat)	1	Structural polymer	Alkylresorcinols, steryl ferulates, lignin-associated phenolics, benzoxazinoids, bound phenolics	Phenolic acids: 200–400 μg/g; Alkylresorcinols: 500–1500 μg/g	Alkylresorcinols serve as biomarkers of whole-grain intake; antimicrobial and antioxidant properties	[17,35,36,52]
Outer pericarp	3–5	Fiber-rich cell wall	Cellulose, hemicellulose, lignin, ferulic acid (75–93%), *p*-hydroxybenzoic acid, vanillic acid, syringic acid	Phenolic acids: 500–950 μg/g; dietary fiber: 60–80%	Highest concentration of bound phenolics; requires colonic fermentation for bioactivation; antioxidant effects in colon	[17,35,36,53]
Whole grain (total)	100	All fractions combined	Complete profile of all above compounds	Total phenolics: 1.4–2.4 mg GAE/g; dietary fiber: 10–15%; protein: 8–15%; minerals: 1.5–2.5%	Comprehensive nutritional and bioactive profile; associated with reduced chronic disease risk	[11,12,13,53]

Abbreviations: SCFAs, short-chain fatty acids; GAE, gallic acid equivalent. Note: Concentration ranges represent typical values reported in the literature; substantial variation exists among wheat varieties, growing conditions, and processing methods. Evidence level: Level 5 (chemical/analytical studies).

**Table 2:** Mineral and Vitamin Content of Wheat Grain Fractions.

	Whole Grain	Bran	Germ	Endosperm (White Flour)	References
**Mineral** (mg/100 g dry weight).					
Potassium (K)	350–450	800–1200	600–900	100–150	[47,48,53]
Phosphorus (P)	250–350	800–1200	400–600	80–120	
Magnesium (Mg)	80–140	300–600	200–300	20–30	
Calcium (Ca)	30–50	80–150	40–60	10–20	
Iron (Fe)	3–5	8–15	5–8	0.5–1.5	
Zinc (Zn)	2–4	5–10	3–5	0.5–1.0	
Copper (Cu)	0.3–0.6	0.5–1.5	0.3–0.6	0.1–0.2	
Manganese (Mn)	2–5	5–10	3–6	0.3–0.8	
Selenium (Se)	0.02–0.06	0.04–0.12	0.02–0.05	0.01–0.02	
**Vitamin** (mg/100 g dry weight).					
Thiamine (B1)	0.3–0.5	0.7–1.2	1.5–2.5	0.05–0.10	[47,48,53]
Riboflavin (B2)	0.1–0.2	0.3–0.5	0.2–0.4	0.02–0.05	
Niacin (B3)	4–6	15–30	5–8	0.5–1.5	
Pyridoxine (B6)	0.2–0.4	0.5–1.0	0.3–0.6	0.05–0.10	
Folate (B9) (μg/100 g)	30–50	80–150	80–120	10–20	
Vitamin E (α-tocopherol)	0.5–1.5	0.5–1.0	10–30	0.1–0.3	
Vitamin K (μg/100 g)	1–5	2–8	5–15	<1	

**Table 3:** Nutritional and antioxidant properties of wheat varieties and derivatives.

Wheat-Related Samples	Key Phytochemicals	Antioxidant Activity	Evidence Level	References
Colored wheat bran fractions	Bound phenolics (ferulic acid, *p*-coumaric acid)	Darker fractions showed antioxidant activity	Level 5	[22]
Fermented wheat bran	Wheat bran polysaccharides (WBP-1) and fermented wheat bran polysaccharides-1 (FWBP-1)	Increased DPPH, hydroxyl, and superoxide radical scavenging	Level 5	[43]
Wheat bran polysaccharides	Complex carbohydrate composition	DPPH and hydroxyl radical scavenging	Level 5	[44]
Durum, soft, red wheat	Red wheat: higher protein, glutamic acid, leucine; minerals (Fe, Zn, Ca)	Red wheat: strongest DPPH scavenging (IC_50_ 121.68 μg/mL)	Level 5	[58]
*Aegilops cylindrica*	Increased polyphenols and flavonoids under salt stress	Increased DPPH and ABTS activities	Level 5	[59]
Wheat aleurone (fermented)	Modified molecular weight, enhanced functional groups	Elevates DPPH and hydroxyl radical scavenging	Level 5	[60]
Wheat germ protein	Peptides YFGWPGPK, FGWPGPR, GHHWPLPP	Strong antioxidant and radical scavenging activity; protected PC12 cells	Level 4	[62]
Black wheat bran (steam explosion)	Improved soluble fiber, phenolics, and flavonoids	DPPH 37.5%, ABTS 31.83%, FRAP 45.82%	Level 5	[65]
Germinated wheat (ultrasound)	Increased GABA (18 mg/100 g), flavonoids (0.19 mg QE/g)	Increased antioxidant capacity (2.86 mg/g)	Level 5	[61]
Wheat sprouts (phytohormone-treated)	Enhanced phenolics, altered metabolite profiles	Improved SOD, CAT, and peroxidase activities	Level 4	[69]

Abbreviations: DPPH, 2,2-diphenyl-1-picrylhydrazyl; ABTS, 2,2′-azino-bis(3-ethylbenzothiazoline-6-sulfonic acid); FRAP, ferric reducing antioxidant power; GABA, gamma-aminobutyric acid; QE, quercetin equivalent; SOD, superoxide dismutase; CAT, catalase; IC50, half maximal inhibitory concentration. Evidence level classification: Level 4 = cell-based experiments; Level 5 = chemical/analytical studies. No human clinical evidence for systemic antioxidant effects is currently available.

**Table 4:** Antidiabetic effects of wheat-derived compounds in preclinical models.

Model	Treatment	Key Mechanism or Outcome	Evidence Level	Limitations	References
HFD/STZ induced T2DM rats	Black wheat chapatti (100 ppm), white wheat chapatti (4 ppm)	Inhibited alpha-amylase and alpha-glucosidase; increased glucose uptake and fatty acid oxidation; enhanced insulin signaling	Level 3	Combined HFD/STZ model; dose may exceed achievable human intake	[76]
Diet/STZ-induced diabetic mice	NSPs from wheat beer (100, 200, 400 mg/kg body weight)	Decreased blood glucose; improved lipid profiles (decreased TC, TG, LDL-C; increased HDL-C)	Level 3	STZ model; acute intervention; limited human relevance	[80]
HepG2 cells	Wheat bran anthocyanins (100, 200, 400 μg/mL)	Increased glucose uptake; increased AKT (Ser473) phosphorylation	Level 4	Cell line model; does not reflect whole-body glucose metabolism	[81]
STZ-induced diabetic Sprague Dawley rats	Whole wheat noodle powder (30, 45, 60 g)	Upregulated liver IRS, IRS-2, GLUT2, AKT, PDK1; enhanced glucose uptake	Level 3	STZ model; dietary intervention, not purified compound	[82]
Insulin-resistant HepG2 cells	Wheat germ peptide	Decreased SOCS3, FOXO1, G6P, phosphoenolpyruvate carboxykinase; increased GLUT2, p-AKT/AKT, p-GSK3β	Level 4	Cell line model; peptide concentration and purity not specified	[83]

Abbreviations: AKT, protein kinase B; STZ, streptozotocin; T2DM, type 2 diabetes mellitus; HFD, high fat diet; TC, total cholesterol; TG, triglycerides; LDL-C, low density lipoprotein cholesterol; HDL-C, high density lipoprotein cholesterol; IRS, insulin receptor substrate; GLUT, glucose transporter; PDK1, pyruvate dehydrogenase kinase 1; SOCS3, suppressor of cytokine signaling 3; FOXO1, forkhead box protein O1; G6P, glucose-6-phosphatase; GSK3β, glycogen synthase kinase 3 beta; NSPs, non-starch polysaccharides. Evidence level classification: Level 3 = animal studies; Level 4 = cell-based experiments.

**Table 5:** Anti-obesity effects of wheat-derived compounds in preclinical models.

Model	Treatment	Key Mechanism or Outcome	Evidence Level	Dose Consideration	Limitations	References
HFD-induced obese C57BL/6 mice	Wheat bran (5%) + oral CbXyn10C xylanase	Reduced body weight and lipid accumulation, improved glycolipid metabolism, reduced systemic inflammation	Level 3	5% of the wheat bran extract oral CbXyn10C orl dose	Short-term preclinical evidence; effects of CbXyn10C and wheat-bran components cannot be completely separated	[90]
HFD-induced obese Sprague Dawley rats	Wheat bran oil (1.25, 2.5, and 5 mL/kg body weight)	Reduced body weight gain and fat and lipid accumulation. Altered gut microbiota composition; decreased Bacteroidota; increased Firmicutes and Proteobacteria	Level 3	Dose (5 mL/kg) exceeds achievable human intake from dietary wheat bran	Animal model; acute intervention; human translation uncertain	[92]
HFD-induced obese rats	Fermented wheat germ (884 mg/kg)	Reduced body weight and alleviated dyslipidemia, inflammation, and leptin resistance	Level 3	Dose (884 mg/kg) exceeds achievable human intake	Animal model; fermented extract not whole food	[93]
HFD-induced obese rats	Wheat germ	Reduced body weight, iWAT and gWAT indices, placental MDA, ROS, and TNF-α levels; improved nutrient transport	Level 3	Dietary intervention; dose not clearly specified	Animal model; pregnancy-specific model	[94]
HFD-induced C57BL/6J mice	Wheat alkylresorcinols (4 g/kg)	Regulated miR-34a and SIRT1 axis, inhibited miR-34a, and increased SIRT1 expression	Level 3	Dose (4 g/kg) far exceeds achievable human intake	Animal model; purified compound; human relevance uncertain	[95]
HFD-induced obese C57BL/6 mice	Wheat peptides (50, 250, and 1000 mg/kg)	Modulated lipid and tryptophan metabolic pathways and inhibited DPP-IV	Level 3	Dose (1000 mg/kg) exceeds achievable human intake	Animal model; peptide mixture composition not fully characterized	[96]
Adults with obesity and diabetes mellitus	Conventional whole-wheat bread or hydroxytyrosol-enriched wheat bread (60 g)	Reduced inflammatory markers and blood lipid levels	Level 1	Human intervention; 60 g bread is a realistic dietary exposure	Small sample size (*n* = 60); 8-week duration; open-label design; no placebo control	[97]
HFD-induced C57BL/6J mice	“Shinmichal” and “Saekeumkang” wheat extracts (100 mg/kg/day)	Reduced adipose and hepatic lipid accumulation	Level 3	Dose (100 mg/kg) exceeds achievable human intake	Animal model; cultivar-specific effects	[98]
Obesity-induced rats	Wheat lipophilic extracts (200 mg/kg/day)	Anti-adipogenic effects via the Wnt and beta-catenin signaling pathway	Level 3	Dose (200 mg/kg) exceeds achievable human intake	Animal model; purified extract not whole food	[100]

Abbreviations: HFD, high-fat diet; iWAT, inguinal white adipose tissue; gWAT, gonadal white adipose tissue; MDA, malondialdehyde; ROS, reactive oxygen species; TNF-α, tumor necrosis factor alpha; miR-34a, microRNA 34a; SIRT1, silent information regulator 1; DPP-IV, dipeptidyl peptidase IV; HbA1c, hemoglobin A1c. Evidence level classification: Level 1 = randomized controlled trials (human); Level 3 = animal studies. Note: Animal doses in this table are reported as administered in the cited studies and should not be directly extrapolated to human dietary exposure. Many doses exceed achievable intake from whole-food wheat consumption.

**Table 6:** Anti-inflammatory effects of wheat-derived compounds.

Model	Treatment	Key Mechanism or Outcome	Evidence Level	Limitations	References
LPS-induced RAW264.7 macrophages	Wheat hull extracts (50 μg/mL)	Reduced nitric oxide production	Level 4	Cell line model; extract from gamma-irradiated mutant lines	[106]
Aged control (50-week-old C57BL/6J mice)	Wheat seedling extract (100, 200, and 400 mg/kg)	Reduced TNF-induced inflammation and improved sarcopenia by regulating protein homeostasis	Level 3	Animal model; aged-specific effects; human translation uncertain	[110]
LPS-induced RAW264.7 cells and rat model	Xianju wheat paste (40 μg/mL)	Reduced iNOS and COX-2 expression and inflammation	Level 4	Cell line and animal model; extract composition complex	[111]
DSS-induced C57BL/6 mice	Wheat bran ingredients	Reduced inflammation and modulated gut microbiota composition	Level 3	Animal model; ingredient composition not fully specified	[112]
Ethanol-induced hepatorenal injury in rats	Wheat germ oil (1.5 mL/kg or 1400 mg/kg body weight orally, for 15 days)	Reduced NF-κB and KIM-1; increased Nrf2 and HO-1; restored antioxidant and immune responses; modulated caspase-3 and Bcl-2 expression	Level 3	Animal model; oil dose exceeds achievable human intake	[113]
LPS-induced Caco-2 cells	Wheat peptides (20, 40, 60, and 80 μg/mL for 24 h)	Inhibited MAPK and NF-κB p65 signaling and reduced inflammatory mediators	Level 4	Cell line model; peptide composition not fully characterized	[116]
Dexamethasone-induced C2C12 myotubes and mouse model	Arriheuk wheat sprouts (500 and 1000 mg/kg body weight for 24 days)	Anti-inflammatory effects mediated by AMPK, FOXO3, and mTOR signaling	Level 3 (in vivo), Level 4 (in vitro)	Combined in vitro/in vivo; dose exceeds achievable human intake	[117]

Abbreviations: TNF-α, tumor necrosis factor alpha; AMPK, AMP-activated protein kinase; FOXO3, forkhead box O3; mTOR, mammalian target of rapamycin; LPS, lipopolysaccharide; MAPK, mitogen-activated protein kinase; NF-κB, nuclear factor kappa B; iNOS, inducible nitric oxide synthase; COX-2, cyclooxygenase 2; DSS, dextran sulfate sodium; KIM-1, kidney injury molecule 1; Nrf2, nuclear factor erythroid 2 related factor 2; HO-1, heme oxygenase 1; Bcl-2, B-cell lymphoma 2. Evidence level classification: Level 3 = animal studies; Level 4 = cell-based experiments.

**Table 7:** Key studies on wheat-derived modulation of the gut microbiota.

Model	Wheat Component	Key Microbiome Changes	Functional Outcome	Evidence Level	Limitations	References
In vitro (SHIME model)	Hydrolyzed and fermented arabinoxylan oligosaccharides	Increased SCFA production and Lactobacillus abundance	Enhanced prebiotic effects compared to non-hydrolyzed bran	Level 4	In vitro model; human translation uncertain	[50]
HFD-induced diabetic mice model	Wheat bran dietary fiber	Increased *Akkermansia muciniphila* abundance; decreased Firmicutes-to-Bacteroidetes ratio	Improved insulin receptor activity and signal transduction	Level 3	Animal model; F/B ratio not universal marker	[140]
Mouse model	Wheat-derived arabinoxylan	Increased colonic regulatory T-cell abundance; enhanced mucosal IgA responses	Strengthened gut barrier and immune function	Level 3	Animal model; human translation uncertain	[141]
In vitro (Caco-2)	Wheat germ protein peptides (VPIPNPSGDR, VPY, AR)	Anti-adhesive activity against Helicobacter pylori	ACE and DPP-IV inhibition	Level 4	In vitro only; peptide purity not specified	[142]
Pregnant sows	Fine-ground wheat bran	Increased *Lactobacillaceae* abundance; increased SCFA, propionate, and butyrate concentrations	Improved satiety, metabolic, and inflammatory status	Level 3	Animal model; pregnancy-specific	[56,143]
Children with wheat allergy	Whole wheat (clinical observation)	Increased *Firmicutes* and *Verrucomicrobia* abundances; shifts in Anaerostipes, Prevotella, and Ruminiclostridium	Altered arginine and polyamine biosynthesis	Level 2	Observational; association not causation	[144]
Older adults	Wheat bran arabinoxylan oligosaccharides	Bifidogenic response associated with baseline Prevotella abundance	Supports personalized prebiotic strategies	Level 2	Small sample; inter-individual variation	[51]
HFD-fed C57BL/6J mice	Whole-wheat and aleurone-enriched flours	Altered gut microbial composition	Modulated hepatic metabolism	Level 3	Animal model; dietary intervention	[145]
Rat model	Red wheat, aleurone, and testa layers	Altered *Lactobacillus, Mucispirillum*, *Phascolarctobacterium*, and *Blautia coccoides*	Influenced colon cancer-related biomarkers	Level 3	Animal model; biomarker endpoints	[146]
In vitro (colon-on-a-plate model)	Whole-grain sourdough bread	Increased SCFA production; altered beneficial microbial taxa	Enhanced effects compared to refined yeast bread	Level 4	In vitro model; donor-dependent variation	[57]

Abbreviations: HFD, high-fat diet; IgA, immunoglobulin A; ACE, angiotensin-converting enzyme; DPP-IV, dipeptidyl peptidase IV; SCFA, short-chain fatty acid; SHIME, simulated human intestinal microbial ecosystem.

**Table 8:** Clinical trials on wheat-derived bioactive compounds.

Intervention	Population	Sample Size	Duration	Key Outcomes	Effect Estimate	Evidence Level	Limitations	References
Whole-grain wheat products	Adults with obesity	*n* = 120	12 weeks	Improved weight loss and cardiometabolic risk factors	Weight loss: −2.1 kg vs. −0.8 kg (control); *p* < 0.05	Level 1	Moderate sample; dietary counseling may confound	[22]
Hydroxytyrosol-enriched wheat bread	Patients with overweight/obesity and T2DM	*n* = 60	8 weeks	Reduced HbA1c, fasting glucose, and body fat mass	HbA1c: −0.4% vs. −0.1% (control); *p* < 0.05	Level 1	Small sample; open-label; bread composition complex	[97]
Wheat aleurone-enriched diet	Adults with abdominal obesity and additional metabolic syndrome features	*n* = 23	8 weeks	Increased postprandial butyrate and reduced oxidative stress	Butyrate: +2.3-fold; MDA: −28%; *p* < 0.05	Level 1	Small sample; short duration; crossover design	[99]
FWGE	Patients with castration-resistant prostate cancer	*n* = 30	12 months	Supportive benefits as adjunct therapy	PSA stabilization in 43% of patients; no adverse events reported	Level 1	Pilot trial; small sample; open-label; no placebo	[137]
Whole-grain wheat biscuits with plant protein	Adults with overweight or obesity	*n* = 45	12 weeks	Reduced body weight, body fat, and TNF-α levels	Weight loss: −3.2 kg vs. −1.8 kg (control); *p* < 0.05	Level 1	Small sample; open-label; no placebo control	[149]

Abbreviations: TNF-α, tumor necrosis factor alpha; T2DM, type 2 diabetes mellitus; HbA1c, hemoglobin A1c; FWGE, fermented wheat germ extract; PSA, prostate-specific antigen; MDA, malondialdehyde. Evidence level classification: Level 1 = randomized controlled trials.

**Table 9:** Molecular targets of wheat bioactive compounds across health outcomes.

Molecular Pathway	Primary Function	Wheat-Derived Activators/Inhibitors	Associated Health Outcomes	Evidence Level	Key References
Nrf2/ARE	Antioxidant defense; phase II enzyme induction	Ferulic acid, caffeic acid, anthocyanins, wheat germ oil	Antioxidant, anti-inflammatory, chemo preventive	Level 3, 4	[60,109,113]
NF-κB	Pro-inflammatory gene transcription	Ferulic acid, alkylresorcinols, wheat peptides, wheat bran polyphenols	Anti-inflammatory, metabolic health, gut barrier protection	Level 3, 4	[109,111,116]
AMPK	Cellular energy sensing; metabolic homeostasis	Anthocyanins, dietary fibers, alkylresorcinols, wheat peptides	Antidiabetic, anti-obesity, anti-sarcopenia	Level 3, 4	[76,95,110]
PI3K/AKT	Insulin signaling; cell survival	Anthocyanins, wheat germ peptides, wheat bran phenolics	Antidiabetic, insulin sensitization	Level 3, 4	[76,81,82,83]
MAPK (ERK/JNK/p38)	Stress response; inflammation	Wheat peptides, wheat bran polyphenols, ferulic acid	Anti-inflammatory, anticancer (cell cycle arrest)	Level 3, 4	[109,116,128]

Evidence level classification: Level 3 = animal studies; Level 4 = cell-based experiments.

**Table 10:** Overview of AI-driven chemical profiling studies in wheat.

Methods	Key Contribution	Sample Size	Validation Approach	Key Limitations	Evidence Level	References
ML-QSAR + genetic algorithm + molecular docking	Predicted phenolic acid-cyclodextrin binding (R^2^ = 0.969–0.984)	20 phenolic acids	Training/testing split (80/20)	Small dataset; computational prediction only	Level 6	[155]
Vis-NIR-SWIR + RF/KNN/DT + XAI	Classified phenolic content with 0.98–0.99 accuracy	Limited number of flour types	Internal validation	Limited external validation; small sample set	Level 6	[156]
RF + WGCNA + multi-omics	Identified naringenin and flavonoid biosynthesis as heat-tolerance biomarkers	Two cultivars	Internal validation	Only two cultivars studied; computational prediction	Level 6	[157]
XGBoost + DNN + portable IoT NIR device	Detected gluten with 94.52% accuracy; testing time <1 min	12,000 samples (corn, rye, oat)	Internal validation	Tested rye, corn, and oat rather than wheat-specific samples	Level 6	[158]
Stepwise linear regression + VIS-NIR + pix2pix GAN	Predicted >220 nutritional components across 402 accessions; visualized spatial distribution	402 global accessions	Internal validation	Wavelength range limited to 1700 nm	Level 6	[159]
RF/DT/KNN + hyperspectral imaging + FTIR	Classified gluten strength with >0.99 accuracy; validated with seven commercial flour blends	Not specified	Internal + limited external (7 blends)	Limited external validation; only three strength categories	Level 6	[160]
SVR + portable NIRS + iWOA wavelength selection	Quantified dry/wet gluten and gluten index using 25–30 wavelengths	Not specified	Internal validation	Requires further field validation	Level 6	[161]
Multi-output ANN + R Shiny interface	Enabled real-time prediction of phenolics, proline, nutrients	Single wheat variety	Internal validation	Single wheat variety tested	Level 6	[162]
LGAKNet (Ghost attention + KAN) + NIR	Predicted protein (R^2^ = 0.9653) and moisture (R^2^ = 0.9683) at 7 samples/s	519 samples	Internal validation	Limited to protein and moisture only	Level 6	[163]
RF regression + Vis-NIR hyperspectral	Predicted wet gluten content (R^2^ = 0.8579)	100 samples	Internal validation	Single growing region; small sample	Level 6	[164]
ML-based peptide predictors + molecular docking	Identified six umami peptides from fermented wheat gluten hydrolysates	Fermented hydrolysate	In silico + sensory testing	Sensory validation limited to in vitro testing	Level 6	[165]
DL + virtual screening + molecular dynamics	Identified gliadin-derived peptide RLSYQFPFYP (Kd = 74.4 nM) with anti-inflammatory activity	2798 peptides screened	In silico + limited in vitro	Screened only 2798 peptides from a single protein	Level 6	[148]

## Data Availability

No new data were created or analyzed in this study. Data sharing is not applicable to this article.

## Supplementary Materials

The following supporting information can be downloaded at: https://www.mdpi.com/article/10.3390/ijms27188290/s1.

## Abbreviations

The following abbreviations are used in this manuscript:

Abbreviation	Full Form
ABTS	2,2’-Azino-bis(3-ethylbenzothiazoline-6-sulfonic acid)
ACE	Angiotensin-converting enzyme
AI	Artificial intelligence
AKT	Protein kinase B
AMPK	AMP-activated protein kinase
ANN	Artificial neural network
ARE	Antioxidant response element
ATM	Ataxia telangiectasia mutated
ATR	Ataxia telangiectasia and Rad3-related
Bax	Bcl-2-associated X protein
Bcl-2	B-cell lymphoma 2
CAM	Class activation mapping
CAT	Catalase
CDK	Cyclin-dependent kinase
CeD	Celiac disease
Chk1/2	Checkpoint kinases 1 and 2
COX-2	Cyclooxygenase 2
CRC	Colorectal cancer
DAG	1,2-Diacylglycerol
DL	Deep learning
DM	Diabetes mellitus
DNN	Deep neural network
DPP-IV	Dipeptidyl peptidase IV
DPPH	2,2-Diphenyl-1-picrylhydrazyl
DSS	Dextran sulfate sodium
DT	Decision tree
DW	Dry weight
EMA	Endomysial antibody
ERK	Extracellular signal-regulated kinase
FAD	Flavin adenine dinucleotide
FAO	Food and Agriculture Organization
FFA	Free fatty acids
FOXO1	Forkhead box protein O1
FOXO3	Forkhead box protein O3
FRAP	Ferric reducing antioxidant power
FTIR	Fourier-transform infrared spectroscopy
FWGE	Fermented wheat germ extract
G6P	Glucose-6-phosphatase
GABA	Gamma-aminobutyric acid
GAE	Gallic acid equivalent
GAN	Generative adversarial network
GC-MS	Gas chromatography-mass spectrometry
GI	Gluten index
GLP-1	Glucagon-like peptide-1
GLUT	Glucose transporter
GMO	Genetically modified organism
GRD	Gluten-related disorder
GSH-Px	Glutathione peroxidase
GSK-3β	Glycogen synthase kinase 3 beta
gWAT	Gonadal white adipose tissue
HBA	Hydroxybenzoic acid
HbA1c	Hemoglobin A1c
HCA	Hydroxycinnamic acid
HDL-C	High-density lipoprotein cholesterol
HFD	High-fat diet
HLA	Human leukocyte antigen
HO-1	Heme oxygenase 1
HOMA-IR	Homeostatic model assessment of insulin resistance
HPLC	High-performance liquid chromatography
HPP	High-pressure processing
IC_50_	Half maximal inhibitory concentration
IDF	International Diabetes Federation
IgE	Immunoglobulin E
IL-1β	Interleukin 1 beta
IL-6	Interleukin 6
iNOS	Inducible nitric oxide synthase
IoT	Internet of Things
IR	Insulin receptor
IRS	Insulin receptor substrate
iWAT	Inguinal white adipose tissue
iWOA	Improved whale optimization algorithm
JNK	c-Jun N-terminal kinase
KAN	Kolmogorov–Arnold network
Keap1	Kelch-like ECH-associated protein 1
KIM-1	Kidney injury molecule 1
KNN	K-nearest neighbors
LC-MS	Liquid chromatography-mass spectrometry
LDL-C	Low-density lipoprotein cholesterol
LGAKNet	Lightweight Ghost-attention Kolmogorov–Arnold network
LPS	Lipopolysaccharide
MAPK	Mitogen-activated protein kinase
MDA	Malondialdehyde
MetS	Metabolic syndrome
MIC	Minimum inhibitory concentration
miR-34a	MicroRNA 34a
ML	Machine learning
MMP-9	Matrix metalloproteinase-9
MSG	Monosodium glutamate
mTOR	Mechanistic target of rapamycin
NAD	Nicotinamide adenine dinucleotide
NCGS	Non-celiac gluten sensitivity
NF-κB	Nuclear factor kappa B
NIR	Near-infrared
NIRS	Near-infrared spectroscopy
NQO1	NAD(P)H: quinone oxidoreductase 1
Nrf2	Nuclear factor erythroid 2-related factor 2
NSP	Non-starch polysaccharide
PDCAAS	Protein Digestibility-Corrected Amino Acid Score
PDK1	Pyruvate dehydrogenase kinase 1
PEPCK	Phosphoenolpyruvate carboxykinase
PGC-1α	Peroxisome proliferator-activated receptor gamma coactivator 1-alpha
PI3K	Phosphoinositide 3-kinase
pix2pix	Pixel-to-pixel generative adversarial network
PKCε	Protein kinase C epsilon
PLS	Partial least squares
PSA	Prostate-specific antigen
PUFA	Polyunsaturated fatty acid
QE	Quercetin equivalent
QSAR	Quantitative structure–activity relationship
RCT	Randomized controlled trial
RF	Random forest
ROS	Reactive oxygen species
SCFA	Short-chain fatty acid
SHIME	Simulated human intestinal microbial ecosystem
SIRT1	Silent information regulator 1
SIRT3	Sirtuin 3
SOCS3	Suppressor of cytokine signaling 3
SOD	Superoxide dismutase
STZ	Streptozotocin
SVR	Support vector regression
SWIR	Short-wave infrared
T2D	Type 2 diabetes
T2DM	Type 2 diabetes mellitus
TC	Total cholesterol
TG	Triglycerides
TLR4	Toll-like receptor 4
TNF-α	Tumor necrosis factor alpha
TRIPOD	Transparent Reporting of a multivariable prediction model for Individual Prognosis or Diagnosis
tTG	Tissue transglutaminase
VEGF	Vascular endothelial growth factor
Vis	Visible
Vis-NIR	Visible–near-infrared
Vis-NIR-SWIR	Visible–near-infrared–short-wave infrared
WCCPS	Wheat cell culture polysaccharides
WDEIA	Wheat-dependent exercise-induced anaphylaxis
WGCNA	Weighted gene co-expression network analysis
WHO	World Health Organization
XAI	Explainable artificial intelligence
XGBoost	Extreme gradient boosting
16S rDNA	16S ribosomal DNA
