# Supplementary material for: Wheat Bioactive Compounds and Human Health: A Review of Nutraceutical Potential, Molecular Mechanisms and AI-Assisted Functional Food Innovation
Source: Int J Mol Sci. 2026 Sep 17;27(18):8290. doi: 10.3390/ijms27188290 (PMC13607264; doi:10.3390/ijms27188290)
Supplement: Supplementary file 1 [file ijms-27-08290-s001.zip › ijms-4536114-supplementary.pdf]

Table S1. Comparison of phenolic acid content across wheat varieties

Wheat type	Total phenolic content (mg GAE/g)	Dominant phenolic acid	Concentrations (μg/g DW)	Key characteristics	Evidence level*	References
Ancient wheat genotypes	1.8–2.4	Syringic acid, ferulic acid	85–210 (syringic)	Higher levels of tyrosol and resorcinol	Level 5	[15]
Whole-grain wheat flour	1.53	Ferulic acid	350–950	Higher phenolic content than refined flour; bound form predominates	Level 5	[16]
White wheat flour	1.20	Ferulic acid	120–280	Lower phenolic content due to removal of bran and germ	Level 5	[16]
Durum wheat (wholemeal)	1.4-2.1	Ferulic acid, *p*-coumaric acid	280-450 (ferulic)	Genotype and location affect phenolic content	Level 5	[17]
Blue wheat bran	2.5–3.8	Ferulic acid, anthocyanins	450–680	Delphinidin derivatives contribute to pigmentation	Level 5	[18]
Purple wheat bran	2.8–4.2	Ferulic acid, sinapic acid	582 (ferulic), 619 (sinapic)	Genotype-dependent variation; higher antioxidant capacity	Level 5	[23]
Sprouted wheat	2.0-3.2	Ferulic acid (free form)	320-580	Germination increases soluble phenolic fraction	Level 5	[27,34]
Black wheat bran	3.5–5.1	Ferulic acid, cyanidin-3-O-glucoside	520–950	Highest anthocyanin content among colored wheats	Level 5	[38]
Fermented wheat bran	3.8-5.6	Ferulic acid (released)	450-820	Enhanced solubility and bioaccessibility	Level 5	[43]
Pigmented durum wheat	2.5-4.8	Ferulic acid, anthocyanins	400-750 (ferulic)	1.6-8.3-fold higher phenolics than refined flour	Level 5	[45]

Abbreviations: GAE, gallic acid equivalent; DW, dry weight. Evidence level classification: Level 5 = chemical/analytical studies (compositional analysis). This table presents compositional data only; no health outcomes are implied.

Table S2. Comparative essential amino acid composition of wheat protein vs. animal protein sources

	(mg/g protein)						References
**Amino Acid**	**Wheat flour**	**Whole wheat**	**Egg (reference)**	**Cow milk**	**Beef**	**WHO/FAO requirement (adult)**	
Lysine	15-25	20-30	70	78	85	45	
Threonine	25-30	28-33	50	44	40	23	[46,47,53,54]
Methionine + Cysteine	30-40	35-45	55	33	35	22	
Leucine	65-70	68-73	86	95	80	59	
Isoleucine	35-40	38-42	54	52	48	30	
Valine	40-45	42-47	66	62	50	39	
Phenylalanine + Tyrosine	70-80	75-85	93	95	75	38	
Tryptophan	10-12	11-14	17	14	12	6	
Histidine	20-25	22-27	24	27	32	15	

Abbreviation: WHO/FAO, World Health Organization/Food and Agriculture Organization.

Table S3. Comparative *in vivo* efficacy of different wheat fractions and colored wheat varieties in animal models.

Wheat fraction/variety	Model	Dose/duration	Key outcomes compared	Reference
Whole black wheat vs. whole common wheat diet	HFD-fed C57BL/6J mice	100 mg/kg/day, 16 weeks	Black wheat showed greater:<br>- Reduction in serum TC, TG, and FFA<br>- Upregulation of fatty acid β-oxidation genes (*crat*, *acca2*, *lonp2*)	[37]
Black wheat bran vs. common wheat bran (steam-exploded)	*In vitro* antioxidant assays	1.0 MPa, 90s	Black wheat bran showed superior:<br>- DPPH scavenging: 37.5% vs. 22.3%<br>- ABTS scavenging: 31.83% vs. 18.5%<br>- FRAP scavenging: 45.82% vs. 28.6%	[65]
Black wheat chapatti vs. white wheat chapatti	HFD/STZ-induced T2DM rats	100 ppm, 30 days	Black wheat showed greater:<br>- Blood glucose reduction: 42% vs. 18%<br>- HbA1c reduction: 35% vs. 12%<br>- HOMA-IR reduction: 48% vs. 22%<br>- Stronger activation of adiponectin/AMPK/PI3K/AKT pathways	[76]
Purple wheat anthocyanin fraction vs. common wheat extract	HepG2 cells	200 μg/mL, 24 h	Purple wheat showed greater:<br>- Glucose uptake: 2.8-fold vs. 1.4-fold<br>- AKT phosphorylation (Ser473): 3.2-fold vs. 1.6-fold	[81]
Fermented vs. unfermented wheat germ	HFD-induced obese rats	884 mg/kg, 12 weeks	Fermented wheat germ showed greater:<br>- Body weight gain reduction: 28% vs. 15%<br>- Adiposity index reduction: 35% vs. 18%<br>- Inflammatory marker reduction (TNF-α): 42% vs. 22%	[93]
Korean whole wheat cultivars "Shinmichal" and "Saekeumkang"	HFD-induced C57BL/6J mice	100 mg/kg/day, 12 weeks	Both cultivars reduced hepatic lipid accumulation; Shinmichal showed superior reduction in atherosclerosis index and coronary risk factors	[98]
Wheat aleurone-enriched diet vs. refined wheat	Adults with cardiometabolic risk	4 weeks	Aleurone-enriched diet showed:<br>- Increased postprandial butyrate: 2.3-fold<br>- Reduced oxidative stress (MDA): –28%	[99]

Abbreviations: HFD, high-fat diet; STZ, streptozotocin; T2DM, type 2 diabetes mellitus; HbA1c, hemoglobin A1c; HOMA-IR, homeostatic model assessment of insulin resistance; AMPK, AMP-activated protein kinase; PI3K, phosphoinositide 3-kinase; AKT, protein kinase B; DPPH, 2,2-diphenyl-1-picrylhydrazyl; ABTS, 2,2'-azino-bis(3-ethylbenzothiazoline-6-sulfonic acid); FRAP, ferric reducing antioxidant power; TC, total cholesterol; TG, triglycerides; FFA, free fatty acids; MDA, malondialdehyde.

Table S4. Gluten-related disorders: key features and management

Disorder	Prevalence	Pathophysiology	Key symptoms	Diagnostic criteria	Management	Evidence level	References
Celiac disease	~1% globally	Autoimmune; HLA-DQ2/DQ8; tissue transglutaminase-mediated deamidation	Diarrhea, malabsorption, weight loss, anemia, dermatitis herpetiformis	Serology (anti-tTG, anti-EMA); intestinal biopsy; HLA typing	Lifelong strict gluten-free diet	Level 1	[54]
Non-celiac gluten sensitivity	0.5-13%	Uncertain; innate immune activation; increased intestinal permeability	Abdominal pain, bloating, fatigue, headache, brain fog	Exclusion of CeD and wheat allergy; symptom improvement on gluten-free diet	Gluten-free diet (may not require strict avoidance)	Level 2	[150]
Wheat allergy	0.2-1% (children)	IgE-mediated; reaction to gluten, albumin, globulin	Oral allergy syndrome, urticaria, anaphylaxis, WDEIA	Skin prick test; serum specific IgE; oral food challenge	Strict avoidance of wheat; epinephrine for severe reactions	Level 2	[151]
Gluten ataxia	Rare	Autoimmune; antigliadin antibodies	Gait disturbance, limb ataxia, dysarthria	Neurological examination; antigliadin antibodies; exclusion of other causes	Gluten-free diet	Level 2	[54]
Dermatitis herpetiformis	Rare	Autoimmune; IgA deposits in skin	Pruritic vesicular rash on extensor surfaces	Skin biopsy with direct immunofluorescence	Gluten-free diet; dapsone for symptom control	Level 2	[54]

Abbreviations: HLA, human leukocyte antigen; tTG, tissue transglutaminase; EMA, endomysial antibody; IgE, immunoglobulin E; WDEIA, wheat-dependent exercise-induced anaphylaxis; IgA, immunoglobulin A. Evidence level classification: Level 1 = clinical guidelines/RCTs; Level 2 = observational human studies.

Table S5. Effects of food processing on wheat bioactive compounds and gluten: benefits and limitations

Processing method	Effects on phenolic compounds	Effects on gluten	Key benefits	Current limitations	Evidence level	References
Thermal processing (baking, extrusion)	Releases bound phenolics; degrades anthocyanins (40-80% loss); ferulic acid stable (65-85% retention)	Partially denatures; may reduce immunogenicity but not eliminate	Improved extractability; enhanced bread quality; reduced microbial load	Heat-labile compound degradation; Maillard byproducts; variable effects across varieties	Level 5	[24,26,54]
Germination/sprouting	Increases soluble phenolics (2-3 fold), GABA, flavonoids; releases bound ferulic acid	Partial proteolytic degradation; may reduce baking quality	Enhanced antioxidant capacity; improved phenolic profile; enriched bioactive metabolites	Time-consuming (24-96 h); sensitive to conditions; may affect texture/sensory; not scalable for some applications	Level 5	[27,34,61]
Fermentation (sourdough, microbial)	Releases bound phenolics; generates quinones (FWGE); enhances bioavailability	Partial proteolysis; reduces but not eliminates immunogenicity	Enhanced antioxidant, anti-inflammatory effects; improved SCFA production	Requires careful strain selection; variable quality; contamination risk; may alter flavor	Level 5	[43,57,93,120]
Enzymatic hydrolysis (ficin, xylanase, esterase)	Releases feruloyl arabinoxylan oligosaccharides; enhances solubilization	Hydrolyzes gluten; may generate bitter peptides	Precise control; improved bioactivity; enhanced functional properties	Enzyme cost; may require debittering; incomplete hydrolysis; limited scalability	Level 5	[49,50,67]
High-pressure processing (HPP)	Preserves heat-labile compounds; enhances bioaccessibility	Modifies structure; reduces immunogenicity	No thermal degradation; improved functional properties	High equipment cost; limited capacity; variable efficacy	Level 5	[139]
Steam explosion	Increases soluble fiber, phenolics, flavonoids, anthocyanins (up to 45-80% increase)	Modifies structure; may affect dough quality	Enhanced antioxidant activity (FRAP 45.82%); improved nutritional profile	Specialized equipment; optimal parameters needed; potential over-treatment	Level 5	[65]
CRISPR/Cas9 genome editing	May affect phenolic biosynthesis pathways (not fully characterized)	97.7% reduction in gluten; eliminated T-cell epitopes	Potential gluten-free wheat with retained bioactives	Regulatory hurdles; GMO concerns; baking quality optimization needed	Level 6	[152,153]

Abbreviations: FWGE, fermented wheat germ extract; SCFA, short-chain fatty acid; GABA, gamma-aminobutyric acid; GMO, genetically modified organism; FRAP, ferric reducing antioxidant power. **Evidence level classification:** Level 5 = chemical/analytical studies; Level 6 = computational predictions.
